# The impact of the method of extracting metabolic signal from ^1^H-NMR data on the classification of samples: A case study of binning and BATMAN in lung cancer

**DOI:** 10.1371/journal.pone.0211854

**Published:** 2019-02-06

**Authors:** Trishanta Padayachee, Tatsiana Khamiakova, Evelyne Louis, Peter Adriaensens, Tomasz Burzykowski

**Affiliations:** 1 I-BioStat, Hasselt University, Diepenbeek, Belgium; 2 Faculty of Medicine and Life Sciences, Hasselt University, Diepenbeek, Belgium; 3 Applied and Analytical Chemistry, Institute for Materials Research, Hasselt University, Diepenbeek, Belgium; George Washington University, UNITED STATES

## Abstract

Nuclear magnetic resonance (NMR) spectroscopy is a principal analytical technique in metabolomics. Extracting metabolic information from NMR spectra is complex due to the fact that an immense amount of detail on the chemical composition of a biological sample is expressed through a single spectrum. The simplest approach to quantify the signal is through spectral binning which involves subdividing the spectra into regions along the chemical shift axis and integrating the peaks within each region. However, due to overlapping resonance signals, the integration values do not always correspond to the concentrations of specific metabolites. An alternate, more advanced statistical approach is spectral deconvolution. BATMAN (**B**ayesian **A**u**T**omated **M**etabolite **A**nalyser for **N**MR data) performs spectral deconvolution using prior information on the spectral signatures of metabolites. In this way, BATMAN estimates relative metabolic concentrations. In this study, both spectral binning and spectral deconvolution using BATMAN were applied to 400 MHz and 900 MHz NMR spectra of blood plasma samples from lung cancer patients and control subjects. The relative concentrations estimated by BATMAN were compared with the binning integration values in terms of their ability to discriminate between lung cancer patients and controls. For the 400 MHz data, the spectral binning approach provided greater discriminatory power. However, for the 900 MHz data, the relative metabolic concentrations obtained by using BATMAN provided greater predictive power. While spectral binning is computationally advantageous and less laborious, complementary models developed using BATMAN-estimated features can add complementary information regarding the biological interpretation of the data and therefore are clinically useful.

## 1 Introduction

Metabolomics characterizes the small molecule or metabolite composition of cells, tissues, or biofluids (e.g., urine, cerebrospinal fluid, or blood plasma). Metabolites are the intermediates or the end products of virtually all biological processes. Changes that occur in the genome, transcriptome, or proteome are reflected in the metabolome. [1] As such, analyzing the metabolic composition of biological samples has considerable potential for disease diagnosis. [2–4] Metabolic profiling also provides information about patient heterogeneity that could play a pivotal role in personalized medicine. [1]

In this study, the metabolic profile of blood plasma was analyzed. One dimensional (1D) proton-nuclear magnetic resonance (^1^H-NMR) spectroscopy is one of the two most commonly used analytical techniques for measuring the metabolite composition of blood plasma. The other analytical technique, mass spectrometry (MS), is more sensitive than ^1^H-NMR spectroscopy, but requires an extraction step to separate the hydrophilic from the hydrophobic metabolites. ^1^H-NMR spectroscopy is a popular choice as it requires minimal sample preparation and because it is a quantitative and non-destructive (i.e., the biological sample remains intact) technique.

^1^H-NMR spectroscopy exploits the magnetic properties of hydrogen nuclei; that is, in a strong external magnetic field, a short radiofrequency (RF) pulse causes hydrogen nuclei to absorb and subsequently emit electromagnetic (EM) radiation. The frequency of RF radiation that is required to bring hydrogen nuclei into resonance (i.e., the frequency of absorbed and re-emitted radiation), is called the resonance frequency (MHz) and it is influenced by the strength of the magnetic field and the chemical environment of the hydrogen nuclei. Resonating hydrogen nuclei produce a NMR response which is called the free induction decay (FID). The FID (time domain) is Fourier transformed to obtain a ^1^H-NMR spectrum (frequency domain) that is visualized as a series of peaks along a chemical shift axis. The peaks correspond to the resonating hydrogen nuclei. The unit of the chemical shift axis is parts per million (ppm), that is, the difference between the resonance frequency of the hydrogen nucleus of the metabolite and the hydrogen nucleus of a reference compound, divided by the resonance frequency of the reference compound. Each metabolite in the biological sample produces a characteristic spectral signature that is formed by a combination of peaks not necessarily adjacent to each other along the chemical shift axis. Each signature appears with an area under the intensity curve that is proportional to the concentration of the corresponding metabolite in the sample.

The identification and quantification of blood plasma metabolites based on ^1^H-NMR spectra is a challenge for the following reasons:

1. ^1^H-NMR spectrometers have detection limits. Although the number of significantly detectable peaks increases for higher magnetic field strengths, the number of existing plasma metabolites that can be reliably detected and quantified remains rather small (approximately 40-50).
2. More than one metabolite can contribute to a signal at a specific location which further complicates peak identification and metabolite quantification.

Typically, ^1^H-NMR metabolomics of blood plasma is conducted using spectrometers with magnetic field strengths ranging from 9.4 Tesla to 14.1 Tesla, i.e., with proton resonance frequencies ranging from 400 MHz to 600 MHz. Higher-field spectrometers (e.g., 900 MHz spectrometers) produce spectra with improved resolution. The ability to resolve peaks with different chemical shifts increases with field strength. However, higher-field spectrometers are also far more costly. [5]

Spectral binning [6] is a simple and commonly used technique for extracting metabolic signal from NMR spectra. It involves subdividing the spectra into regions along the chemical shift axis and computing the area under the curve within each integration region. The limits of the integration regions are defined by spectroscopists in a way that best accommodates the metabolite peaks of interest. However, peak overlap and variation in the chemical shift positions of the peaks across spectra often prevents a one-to-one mapping between integration regions and metabolites. This may be especially problematic in the context of sample classification. In particular, an integration region may fail to show potential for classification if it includes metabolites which show opposite behavior (under- and over-expression) in patients versus controls. For instance, assume an integration region encompasses signal coming from two discriminative metabolites. On average, one metabolite has a higher concentration in patients than in controls while the second metabolite has a lower concentration in patients than in controls. Despite the fact that the integration region contains signal from two discriminative metabolites, the opposite behavior of the two metabolites diminishes the classification potential of the integration region, potentially resulting in a non-differential integrated spectral region (ISR). On the other hand, for an integration region that shows classification potential it may be difficult to uniquely assign its effect to a single metabolite.

Since overlapping molecular resonances complicate the extraction of metabolic information from ^1^H-NMR data, spectral deconvolution techniques are currently the state of the art. BATMAN (Bayesian AuTomated Metabolite Analyser for NMR data) [7, 8] is a Bayesian model for ^1^H-NMR spectral deconvolution which resolves resonance peaks to obtain relative concentration estimates for a set of metabolites in an automated manner. It exploits extensive prior information on the characteristic resonance signatures of each metabolite and combines this information with the intensities observed in the actual spectrum to model the metabolic signal. Other deconvolution models include Bayesil and the commercially available software package Chenomx amongst others. [9, 10] The advantage of BATMAN is its flexibility and adaptability to the problem at hand. The prior information on peak shape and relative intensity plays an important role in any spectral deconvolution and signal extraction model. Flexibility in setting up the prior information is desirable especially when ^1^H-NMR spectroscopy is performed on a spectrometer different to the one used to create the software.

The spectra, the ISRs obtained through spectral binning, and the relative metabolite concentrations estimated by BATMAN are quantitative measures of the signal. However, the measures are normalized such that the values become relative to a normalization constant (e.g., the total area under the curve of all integration regions).

In this article, the application of the widely used spectral binning approach is compared with the automated spectral deconvolution technique, BATMAN, for extracting metabolic signal for the purposes of sample classification. The two approaches were applied to 400 MHz (medium-field) and 900 MHz (high-field) ^1^H-NMR spectra of blood plasma samples from lung cancer patients and control subjects. The extracted features, that is, the ISRs and the BATMAN estimated relative concentrations of the metabolites, were compared in terms of their ability to correctly classify lung cancer and control samples. This was performed separately for the 400 MHz and 900 MHz spectra.

A series of pre-processing steps were required to reduce the noise, external sources of variation, and artifacts which result during the process of NMR data acquisition before the metabolic signal could be extracted. Different pre-processing protocols were applied to the 400 MHz and 900 MHz ^1^H-NMR spectra. In particular, the use of a more automated approach for pre-processing the 900 MHz ^1^H-NMR spectra was investigated.

## 2 Materials and methods

### 2.1 Data

For this investigation, the previously analyzed [5], ^1^H-NMR spectra of blood plasma samples obtained from lung cancer patients (*n*_cases_ = 69), included in the Limburg Positron Emission Tomography center (Hasselt, Belgium) from March 2011 to January 2012, and control subjects (*n*_controls_ = 74), attending Ziekenhuis Oost-Limburg (Genk, Belgium) between December 2011 and April 2012, were used. There was no drop-out. The following exclusion criteria were applied:

1. not fasted for at least 6 hours,
2. a fasting blood glucose concentration ≥200 mg/dl,
3. medication intake on the morning of blood sampling, and
4. treatment or history of cancer in the past 5 years.

The study was conducted in accordance with the ethical rules of the Helsinki Declaration and Good Clinical Practice and was approved by the ethical committees of Ziekenhuis Oost-Limburg (ZOL) and UHasselt. All study participants provided written informed consent.

Fasting venous blood samples were collected in 10 ml lithium-heparin tubes and stored at 4°C within 5 to 10 minutes. Within 8 hours after blood collection, samples were centrifuged at 1600 g for 15 minutes, and plasma aliquots of 500 *μl* were transferred into sterile cryovials and stored at −80°C until NMR analysis within six months.

The ^1^H-NMR data were acquired by analyzing the blood plasma samples at 21.2°C on a 400 MHz spectrometer (9.4 Tesla; 54 mm bore-size; Varian Inova; Agilent Technologies Inc.; VnmrJ 3.2 RevisionA) and on a 900 MHz spectrometer (21.1 Tesla; 54 mm bore-size; Bruker Avance; Bruker Biospin). The 400 MHz spectrometer is equipped with an Agilent OneNMR 5mm probe, whereas the 900 MHz spectrometer has a triple resonance cryoprobe. Slightly T2-weighted spectra were acquired using the Carr-Purcell-Meiboom-Gill pulse sequence (total spin-echo time of 32 ms; interpulse delay of 0.1 ms), preceded by an initial preparation delay of 0.5 s, and 3 s for water suppression presaturation. Other parameters for acquiring the 400 MHz/900 MHz data, respectively were: a spectral width of 6000 Hz/14423 Hz, a 90° pulse length of 6.35/9.15 μs, an acquisition time of 1.2 s, a preparation delay of 3.5 s, and 96/64 scans (7min 44sec/5min 9sec on 400 MHz/900 MHz).

### 2.2 Spectral pre-processing

A manual pre-processing protocol was applied to the 400 MHz and the 900 MHz spectra. The 900 MHz spectra were also pre-processed using a more automated protocol.

#### 2.2.1 Manual pre-processing

The 400 MHz spectra were pre-processed using the Varian/Agilent software. The pre-processing steps included zero-filling and multiplication by an exponential apodization function of 0.7 Hz prior to the Fourier transformation. The spectra were manually phased, automatically baseline corrected using polynomials (or splines), and referenced to trimethylsilyl-2,2,3,3-tetradeuteropropionic acid (TSP) at 0.015 ppm. [4] The final step of the spectral pre-processing was normalization by the total area under the curve, without accounting for the water and TSP signal.

The 900 MHz Bruker files were first transformed to the Varian format for compatibility with the Varian pre-processing software before being manually pre-processed in the same way as the 400 MHz data.

#### 2.2.2 Automated pre-processing

**Fig 1:**
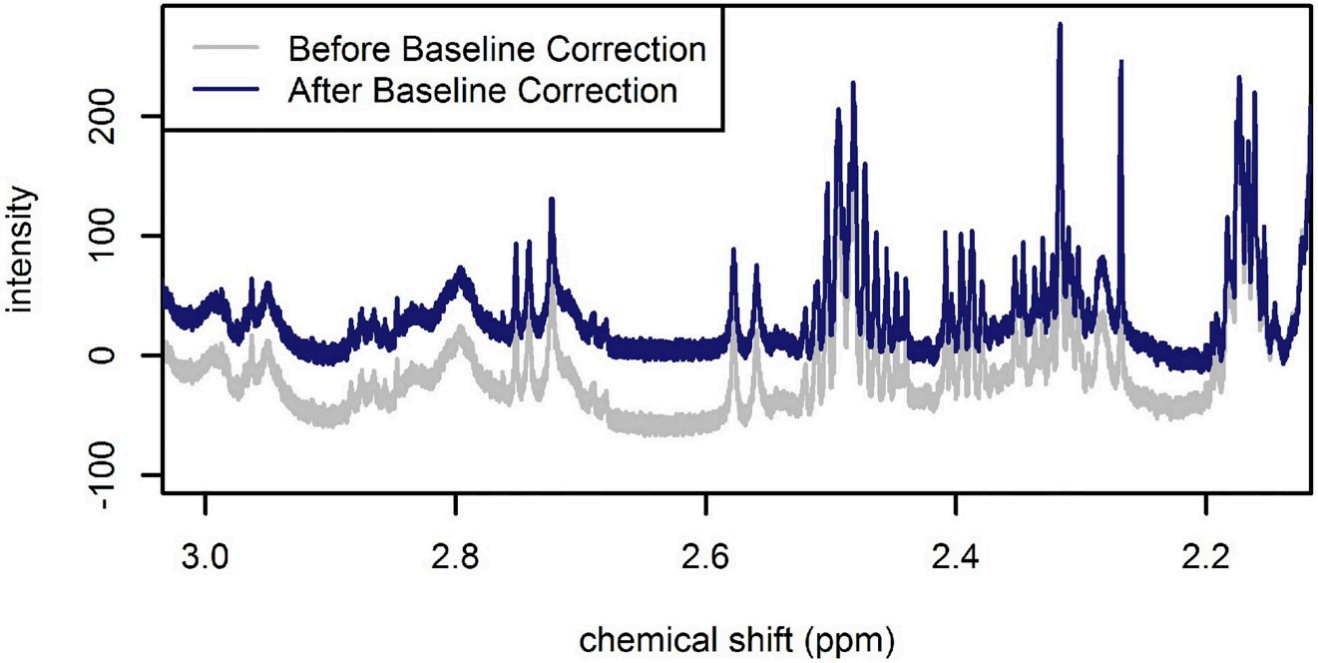
Illustration of a portion of a 900 MHz spectrum before (grey spectrum) and after (blue spectrum) baseline correction.

The 900 MHz spectra were also automatically pre-processed using the R statistical software package PepsNMR [11]. PepsNMR was applied to the raw Bruker FIDs. Pre-processing included a first-order and zero-order phase correction, solvent (i.e., water) suppression, apodization, zero-filling, Fourier transformation, baseline correction, spectral alignment, and median normalization. The default PepsNMR settings were utilized for all steps prior to the baseline correction. A more stringent penalty was selected for the baseline correction in order to keep the number of spectral points with negative intensities to a minimum. That is, the PepsNMR baseline correction asymmetry parameter was set to 0.01 (see Fig 1).

**Fig 2:**
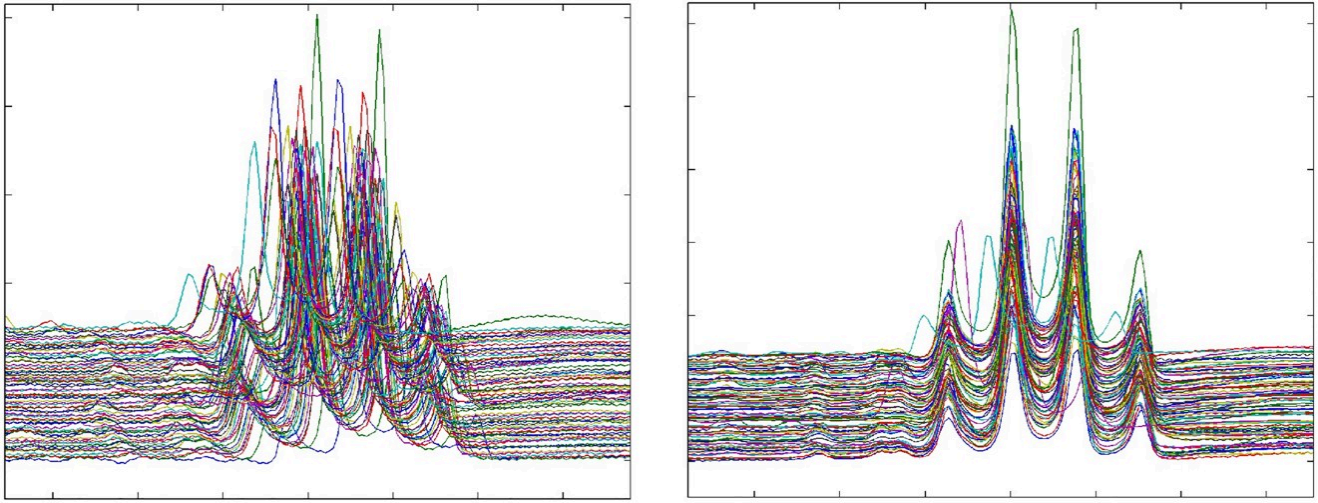
Illustration of warping in the region of the lactate signal. Left: a portion of a 900 MHz spectrum before warping. Right: a portion of a 900 MHz spectrum after warping.

The chosen reference spectrum for spectral alignment was the spectrum that achieved the smallest sum of squared differences between the reference spectrum and all other spectra after warping. This corresponds to setting the reference choosing parameter of the PepsNMR warping function to *after* (see Fig 2 and Fig A in S1 File). Since we expect differences in the metabolic profile between lung cancer patients and control subjects, warping was performed separately for the two groups.

**Fig 3:**
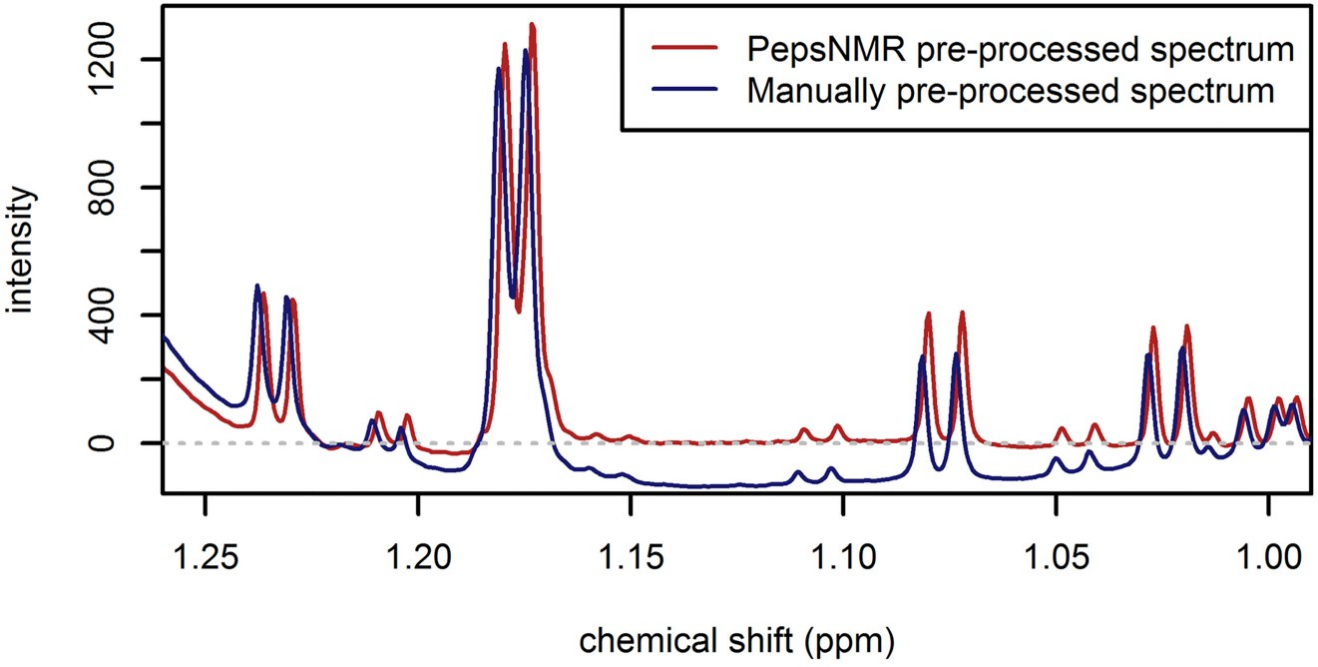
Illustration of a portion of a 900 MHz PepsNMR automatically pre-processed spectrum and a 900 MHz manually pre-processed spectrum.

The main reason for applying two different pre-processing protocols to the 900 MHz spectra was that the manual pre-processing of the 900 MHz spectra did not provide data of sufficient quality to perform the BATMAN analysis (see Fig 3). It was necessary to use the raw FID data to improve the manual baseline correction and to avoid numerous manual steps in phasing. With PepsNMR, the pre-processing steps and parameter settings can be clearly defined which improves the reproducibility of the analysis.

### 2.3 Spiking experiments

Spiking experiments [6] were conducted to determine the chemical shift positions of the blood plasma metabolites. Spiked spectra were acquired on the 400 MHz and 900 MHz spectrometers for 37 metabolites: alanine, arginine, asparagine, aspartate, cysteine, glutamine, glutamate, glycine, histidine, isoleucine, leucine, lysine, methionine, phenylalanine, proline, serine, threonine, tryptophan, tyrosine, valine, glucose, myo-inositol, acetate, acetoacetate, *α*-ketoglutarate, *β*-hydroxybutyrate, citrate, lactate, pyruvate, succinate, creatine, creatinine, acetone, betaine, choline, glycerol, and methanol.

### 2.4 Spectral binning for NMR signal extraction

Spectral binning involves partitioning the ^1^H-NMR spectra into regions along the chemical shift axis. The resonance peaks encompassed by each region are integrated. The resulting ISRs constitute a set of features that represent the NMR signal. Reliable information on the chemical shift of metabolite peaks is essential for the identification of biologically meaningful spectral regions. Using the chemical shift information acquired through spiking experiments, the 400 MHz spectra were subdivided into 110 integration regions of varying widths excluding the water region and the TSP region (see Table A in S1 File) [6]. Similarly, the manually pre-processed 900 MHz spectra were partitioned into 105 integration regions [5] and the PepsNMR automatically pre-processed 900 MHz spectra were partitioned into 103 integration regions (see Table A in S1 File). Further details on the standard protocol for extracting features from NMR data using spectral binning can be found in Louis et al., (2015) [6].

### 2.5 BATMAN

The Bayesian state-of-the-art spectral deconvolution technique, BATMAN, was developed by Astle et al., (2012) [7]. BATMAN resolves the resonance peaks of NMR spectra in order to estimate the relative concentrations of a pre-specified set of metabolites. BATMAN is a two-component model. The first component models the metabolic signal (i.e., the signal assigned to specific metabolites) while the second component models the residual signal. BATMAN exploits extensive prior information on the characteristic spectral signatures of each metabolite and combines this information with the observed intensities to model the metabolic signal. The second component uses wavelets to capture the residual signal. The residual signal includes the signal that arises from other uncatalogued chemical constituents such as lipids. When the metabolic signal has been properly extracted, the wavelet signal can be divided into carefully selected broad integration regions to approximate lipid concentrations. In this way, a set of relative metabolite concentrations and lipid features can be obtained. In addition to providing point estimates of the metabolic concentrations per spectrum, BATMAN also provides 95% credible intervals for each estimate which can be used to assess the degree of uncertainty in the estimated concentrations.

BATMAN was implemented by using the R statistical software package batman, as detailed in the protocol by Hao et al., (2014) [8]. In this section, the implementation of the model is described and some of the steps that are crucial for improving the extraction of the metabolic signal are summarized.

The standard BATMAN inputs are the NMR spectroscopy data (NMRdata.txt), the parameter options file (batmanOptions.txt), the library of characteristic metabolic signatures (multi_data.csv or multi_data_user.csv), and a list of the metabolites of interest (metabolitesList.csv).

#### 2.5.1 BATMAN options file

The parameter settings (batmanOptions.txt) used for the 400 MHz and the 900 MHz analysis are shown in Table B in S1 File.

#### 2.5.2 Template file

Prior information about the spectral signatures of each metabolite is specified in the default BATMAN template file multi_data.csv. The default template file can be modified by constructing the template file multi_data_user.csv. The fit of the BATMAN model can be improved considerably by providing prior information that more accurately describes the observed peaks. Each resonance is described in the BATMAN template file in terms of its chemical shift position (in ppm), multiplicity (i.e., the J-coupling pattern, i.e., whether the signal appears as a singlet, doublet, triplet, or double doublet etc.), J-coupling constants, and the relative intensities of the peaks. Multiplets with well-defined coupling patterns and known coupling constants, that exhibit second order effects (i.e., roofing∖leaning effects), can be modeled empirically by specifying the observed intensity ratios of the peaks (see the note on empirical multiplets below). Complex multiplets (e.g., multiplets that are not well-defined or those that exhibit higher order coupling patterns), for which elucidation would require a substantial amount of input from a spectroscopist, may be modeled as raster multiplets by providing a corresponding section of a pure compound spectrum (see the note on raster multiplets below).

Given the complexity of NMR resonances, ill-defined chemical shift positions is the recipe for a poor fit. Prior information about the peak locations was determined by using the splineFit routine [8] implemented in Matlab and the details on the ^1^H-NMR chemical shift locations of plasma metabolites reported by Louis et al., (2015) [6].

#### 2.5.3 A note on empirical multiplets

For the user-defined empirical multiplets, the accurate specification of relative intensities is subject to the availability of pure compound spectra (i.e., NMR spectra obtained by analyzing a sample containing only the target metabolite). For the multiplets in regions of no overlap, baseline-corrected spiked spectra were used as a substitute for the pure compound spectra. To compute the relative intensities, the number of resonating protons should be taken into account. For a particular multiplet, a simple numeric solution is to take the intensities of the peaks observed in the pure compound spectra and to normalize them to sum to the actual number of protons. That is, the relative intensity of a multiplet’s *i*^th^ peak is computed using the following formula:

$$\begin{array}{c} h_{i}=p\times \frac{y_{i}}{\sum_{i}y_{i}} \end{array}$$ (1)

where *y*_*i*_ is the observed intensity based on pure compound spectra and *p* is the number of protons associated with the multiplet.

**Fig 4:**
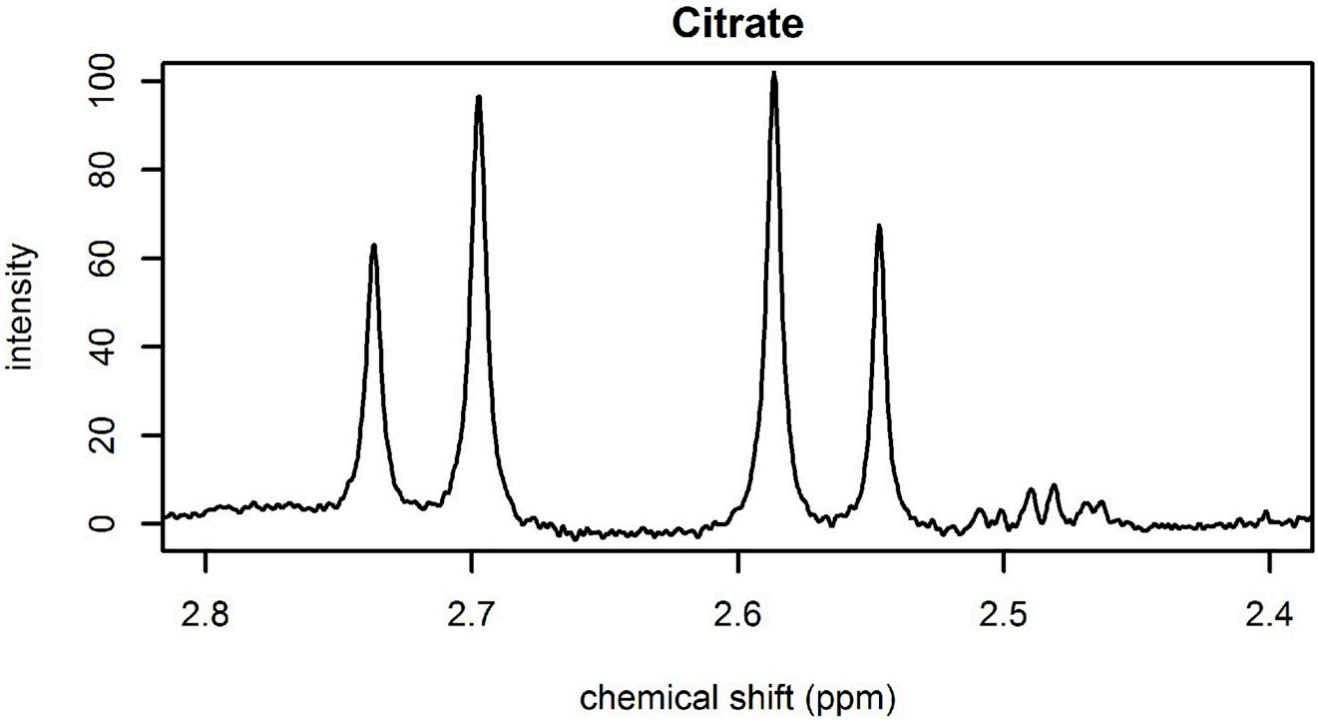
Illustration of a 400 MHz spectrum with two doublets of citrate at 2.717 and 2.566 ppm. Each doublet arises from a CH_2_-group and thus from 2 protons.

Empirical templates can be defined to model multiplets that exhibit roofing∖leaning effects. Fig 4 shows the leaning effect of two doublets of citrate. Each doublet was produced by two resonating protons. Using the equation above, the relative intensity of the peak at 2.586 ppm is *h*_1_ = 1.2 and the relative intensity of the peak at 2.547 ppm is *h*_2_ = 0.8.

**Fig 5:**
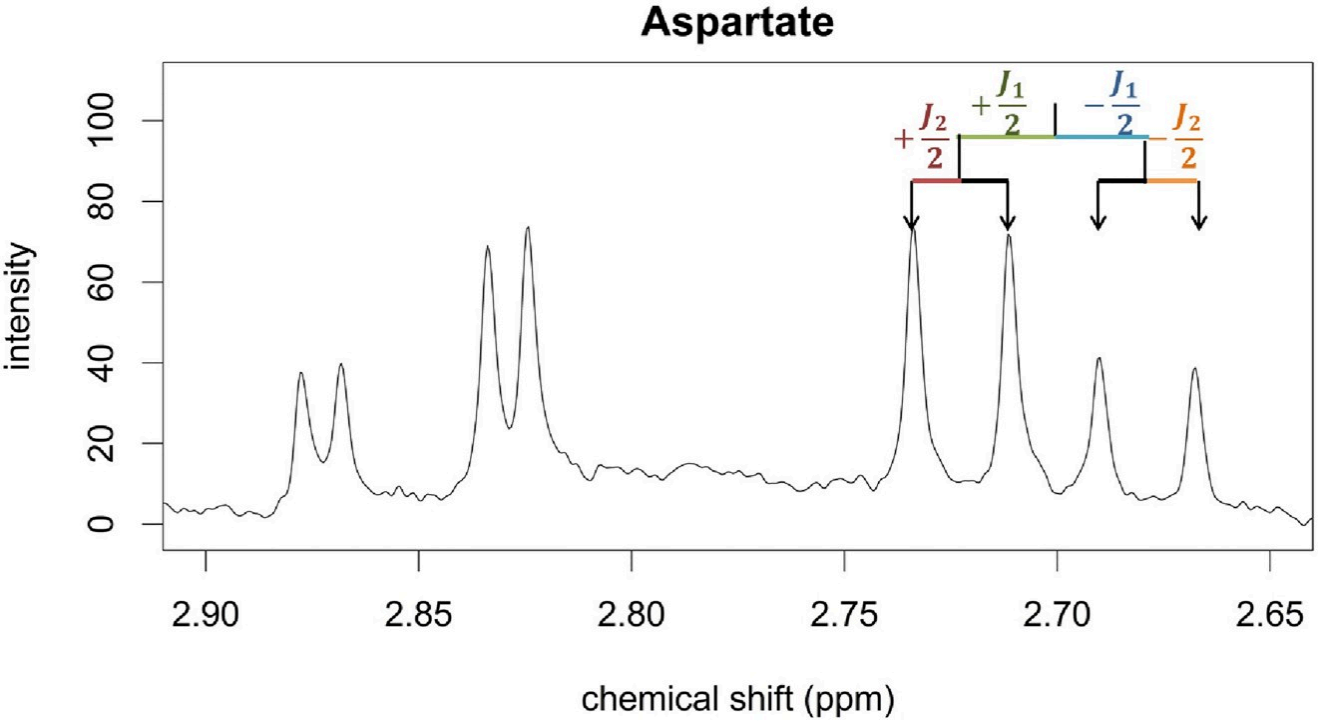
A portion of a 400 MHz spectrum illustrating the identification of peak offsets for the double doublet of aspartate at 2.702 ppm. Coupling constants J_1_ and J_2_ can be used to obtain the location of the four peaks from the center of the multiplet.

In addition to the relative intensities, the offset of the peaks should be specified (in Hz). Offsets are specified from a point of origin. For convenience, the center of the multiplet can be taken as the origin. The offsets can be determined from pure compound spectra. Alternatively, the J-coupling information of ^1^H-NMR plasma metabolites reported by Louis et al., (2015) [6] and public databases like the Human Metabolome Database (HMDB) can be used. This is depicted in Fig 5, where the offset of the leftmost peak from the center of the double doublet of aspartate is half of the sum of the two J-coupling constants.

#### 2.5.4 A note on raster multiplets

**Fig 6:**
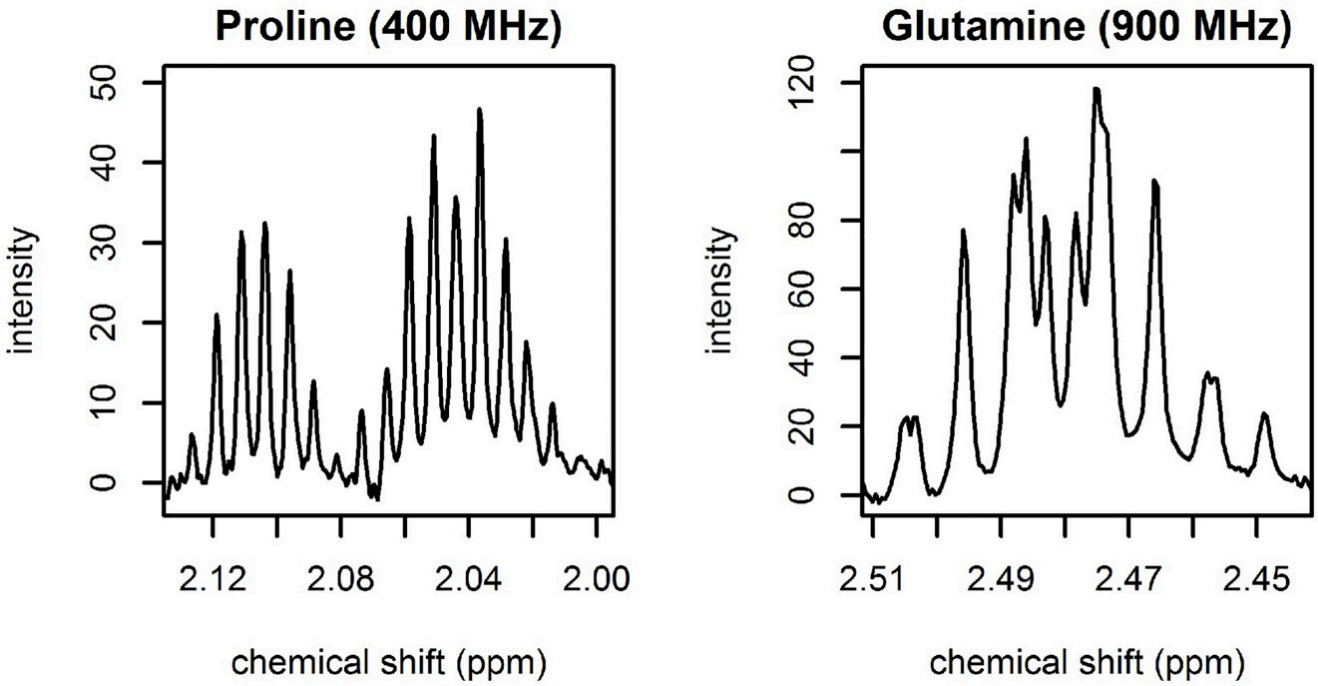
Illustration of raster multiplets. Left: 400 MHz raster multiplet for proline. Right: 900 MHz raster multiplet for glutamine.

Raster multiplets can be modeled from pure compound spectra. Due to the lack of pure compound spectra, spiked spectra were used for the multiplets that are found in regions where there is no significant overlap with other metabolites. Examples of raster multiplets for the 400 MHz and 900 MHz spectra are shown in Fig 6.

#### 2.5.5 Target metabolites

The BATMAN model was applied to estimate the relative concentrations of the following metabolites in the 400 MHz spectra: alanine, arginine, asparagine, aspartate, cysteine, glutamine, glutamate, glycine, histidine, isoleucine, leucine, lysine, methionine, phenylalanine, proline, serine, threonine, tryptophan, tyrosine, valine, *α*-D-glucopyranose, *β*-D-glucopyranose, myo-inositol, acetate, acetoacetate, *α*-ketoglutarate, *β*-hydroxybutyrate, citrate, lactate, pyruvate, succinate, creatine, and creatinine. In the 900 MHz spectra, betaine and choline were added to the above list of metabolites. However, for consistency between the 400 MHz and 900 MHz analysis, Betaine and Choline were not used as classification features. Louis et al., (2015) [6] and Louis et al., (2017) [5] specify the chemical shifts of these metabolites.

#### 2.5.6 Verifying the goodness of the BATMAN fit

**Fig 7:**
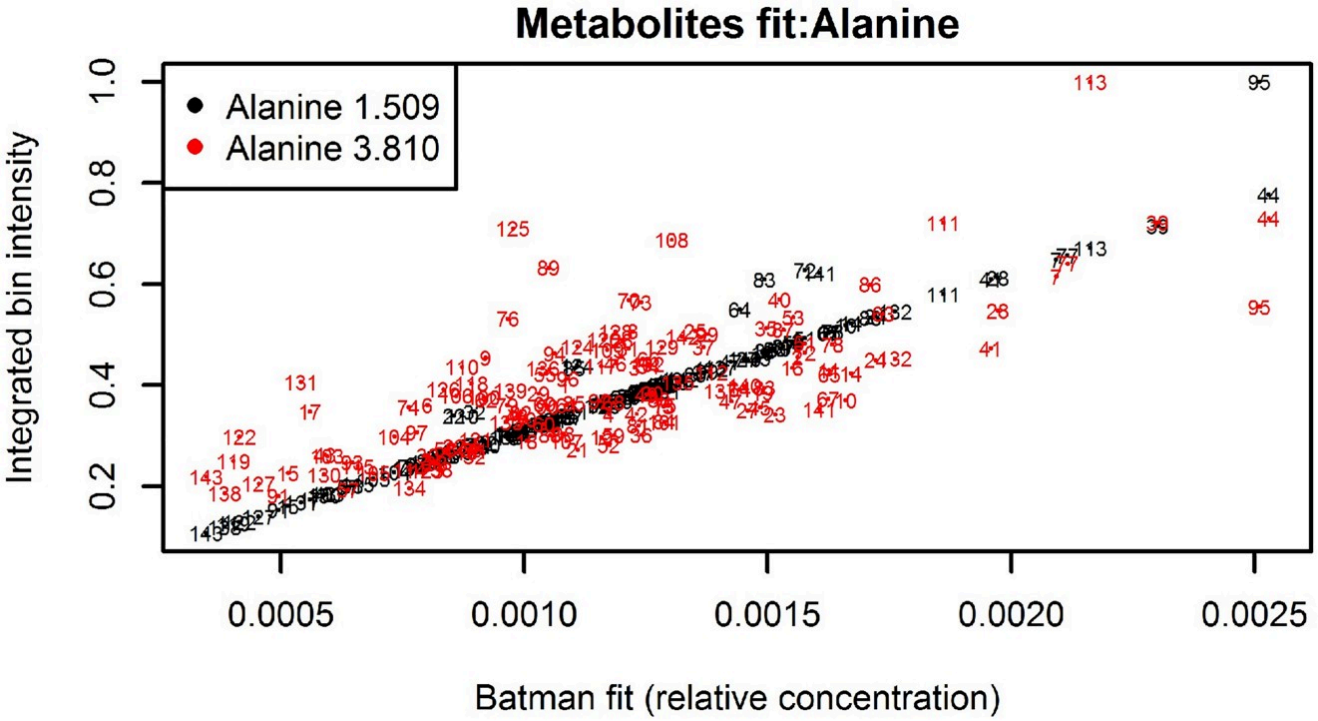
Batman diagnostic plot for alanine. Each number corresponds to a specific spectrum.

The goodness of fit of the modeled metabolic signal can be checked by using the built-in tools of the R batman package. Hao et al., (2014) [8] provided straightforward guidance on the use of the batman diagnostic plots. However, it is worthwhile to note that comparing the integrated bin intensities with the BATMAN metabolite fit for multiplets in crowded-peak regions is less informative (i.e., it is not a solution for evaluating the BATMAN fit or for identifying problem spectra). To illustrate this point, consider Fig 7 showing the diagnostic scatterplot for alanine. While the integration values are aligned for the doublet at 1.509 ppm, they are somewhat scattered for the quadruplet at 3.810 ppm. This is primarily due to the glucose resonances and signals from other metabolites that lie in the vicinity of the quadruplet and which contribute to the integrated bin intensity (see Fig B in S1 File).

#### 2.5.7 Post-processing of spectral fits

**Fig 8:**
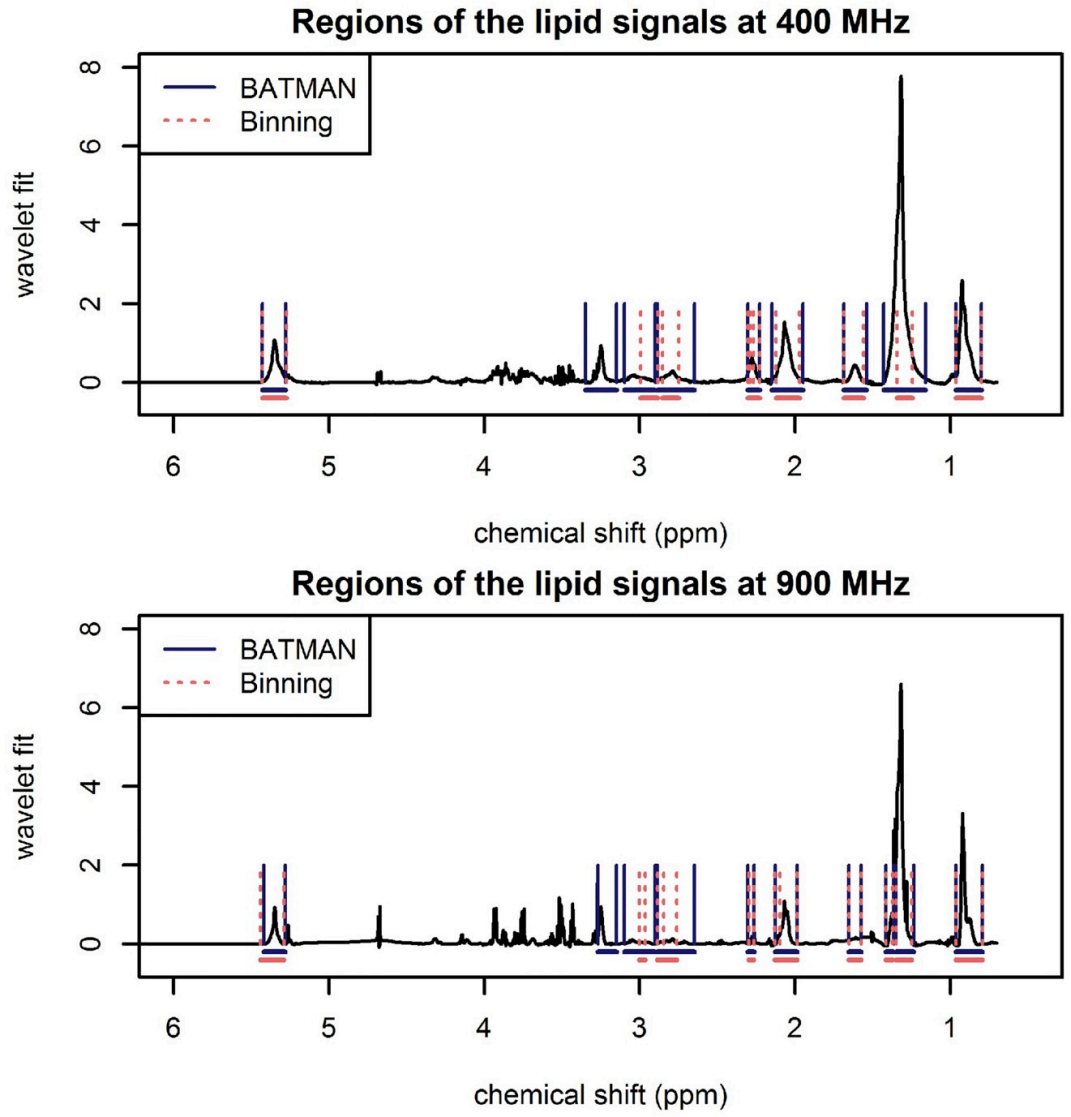
Illustration of the BATMAN wavelet-fit showing lipid integration regions for a 400 MHz (top) and 900 MHz (bottom) spectrum. The BATMAN integration regions that capture the broad lipid resonances are delimited by blue solid lines. The narrower spectral binning integration regions (delimited by red dashed lines) capture lipid signals, but not necessarily exclusively.

Once the metabolic signal has been correctly assigned, the residual signal captured by the wavelet component of the BATMAN model can be used to estimate the lipid concentrations. Towards this aim, integration regions that encompass lipid resonances were specified. Lipid resonances typically appear as broad peaks in NMR spectra. In general, the lipid integration regions selected for the BATMAN analysis are broader than those used for spectral binning (see Fig 8 and Table C in S1 File). The defined integration regions aim to capture the following broad lipid resonances: C**H**_3_–(CH_2_)_n_–in the fatty acid chain (FAC), –CH_3_–(C**H**_2_)_n_–in the FAC (captured using two integration regions in the 900 MHz analysis), –C**H**_2_–CH_2_–C = O or –C**H**_2_–CH_2_–CH = CH–in the FAC, –C**H**_2_–CH = CH–in the FAC and C**H**_3_ in N-acetylated glycoproteins (NAG), –C**H**_2_–C = O or –C**H**_2_–CH = CH–in FAC, = CH–C**H**_2_–CH = in FAC, lysyl, and –C**H** = C**H**–in FAC. In this way, a set of lipid-specific features were obtained in addition to the relative metabolic concentrations. This approach only works when the metabolic signal has been sufficiently extracted. Should this not be the case, the residual signal will be contaminated by other metabolites resonating in the area.

### 2.6 Classification

Spectral binning and spectral deconvolution by BATMAN were applied to the manually pre-processed (mp) 400 MHz and the PepsNMR automatically pre-processed (ap) 900 MHz ^1^H-NMR spectra of lung cancer patients and control subjects. Spectral binning was also applied to the manually pre-processed 900 MHz ^1^H-NMR spectra. As a result, the following five sets of predictors were obtained:

1. Spectral binning ISRs for 110 regions based on the mp 400 MHz spectra.
2. Relative concentrations obtained using BATMAN for 33 metabolites and 9 lipid features based on the mp 400 MHz spectra.
3. Spectral binning ISRs for 103 regions based on the ap 900 MHz spectra.
4. Relative concentrations obtained using BATMAN for 33 metabolites and 10 lipid features based on the ap 900 MHz spectra.
5. Spectral binning ISRs for 105 regions based on the mp 900 MHz spectra.

**Fig 9:**
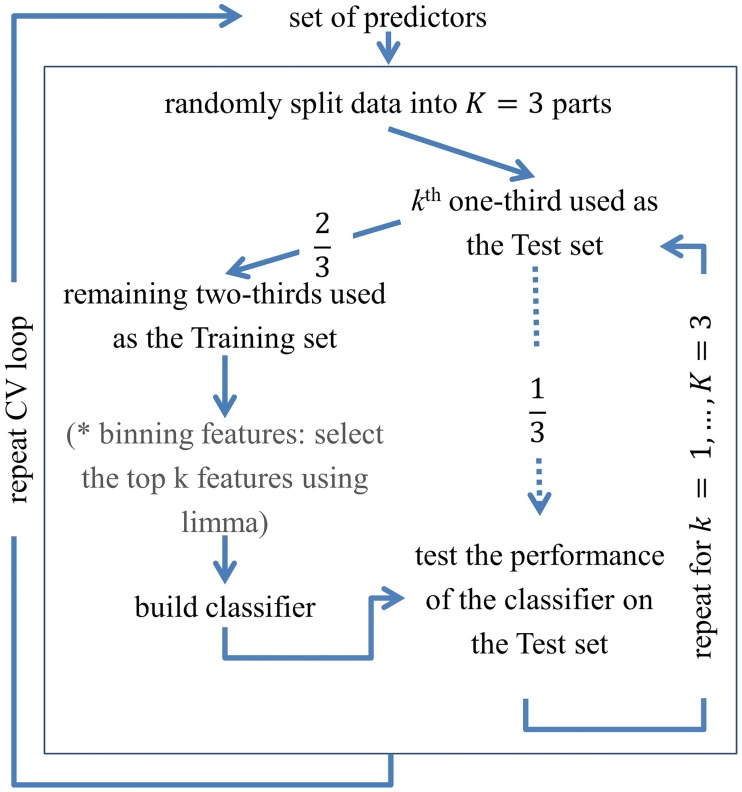
The three-fold cross-validation procedure.

Classifiers were built by using each set of predictors. The predictive performance of the classifiers was assessed by using a three-fold cross-validation (CV) scheme (see Fig 9). CV works by dividing the dataset in two parts, a training set and a test set. In *K*-fold CV, the data are split into *K* roughly equal parts. In the *k*^th^ iteration, where *k* = 1, …, *K*, the *k*^th^ part of the data forms the test set and the remaining *K* − 1 parts form the training set. Thus, in three-fold CV, one-third of the data forms the test set and the remaining two-thirds of the data (i.e., the training set) are used to build the classifier. At each iteration, the performance of the classifier is evaluated in terms of the proportion of misclassifications and the sensitivity and specificity of the classifier when applied to the test set. Since the splitting is not uniquely determined, [12] the cross validation procedure was repeated 333 times. The overall performance is based on the mean classification error rate, the mean sensitivity, and the mean specificity of the 999 classifiers.

For the classification analysis involving the binning features, variable selection was based on the discriminative power of the individual bins between the two conditions. This was assessed by using the limma-based moderated t-statistic [13], as indicated by the asterisk in Fig 9. The classification analysis involving the BATMAN estimated features proceeded using the following three subsets of the features: (1) all the BATMAN-estimated relative metabolite concentrations, (2) all the relative lipid concentrations, and (3) all the BATMAN-estimated relative metabolic concentrations together with all the relative lipid concentrations.

Five procedures that are appropriate for the analysis of large, complex datasets were used to build the classifiers, namely elastic net, lasso, orthogonal partial least squares-discriminant analysis (OPLS-DA), support vector machines (SVMs), and random forests (RF). A brief description of each method is provided in S1 File and the reader is referred to Hastie et al., (2009) [14] and Bylesjö et al., (2006) [15] for further details.

Although the limma-based moderated t-statistic was not used to build the BATMAN feature-based classifiers, the test was applied (in 333 iterations of three-fold cross-validation) as a univariate approach to identify the top 15 variables of each of the five sets of predictors. These variables were identified to check whether there were any similarities in the most discriminative variables selected for each classification task.

The statistical analysis was conducted by using the R statistical software (version 3.2.3, R Development Core Team, 2015). Classification methods were implemented by using the default options of the R Bioconductor package CMA [12].

## 3 Results

For the BATMAN analysis, many multiplets were modelled best by using either empirical multiplets or raster multiplets. However, as stated in [8], while this does not necessarily result in a perfect fit, it does allow the user to capture metabolites, which may otherwise not be possible. Despite extensive adjustments of the BATMAN template file, we did not achieve a perfect fit for all the multiplets across all the spectra (see Figs C and D in S1 File). Due to this, the selected lipid regions sometimes contained residual metabolite peaks (see Figs C, D, E, and F in S1 File).

**Fig 10:**
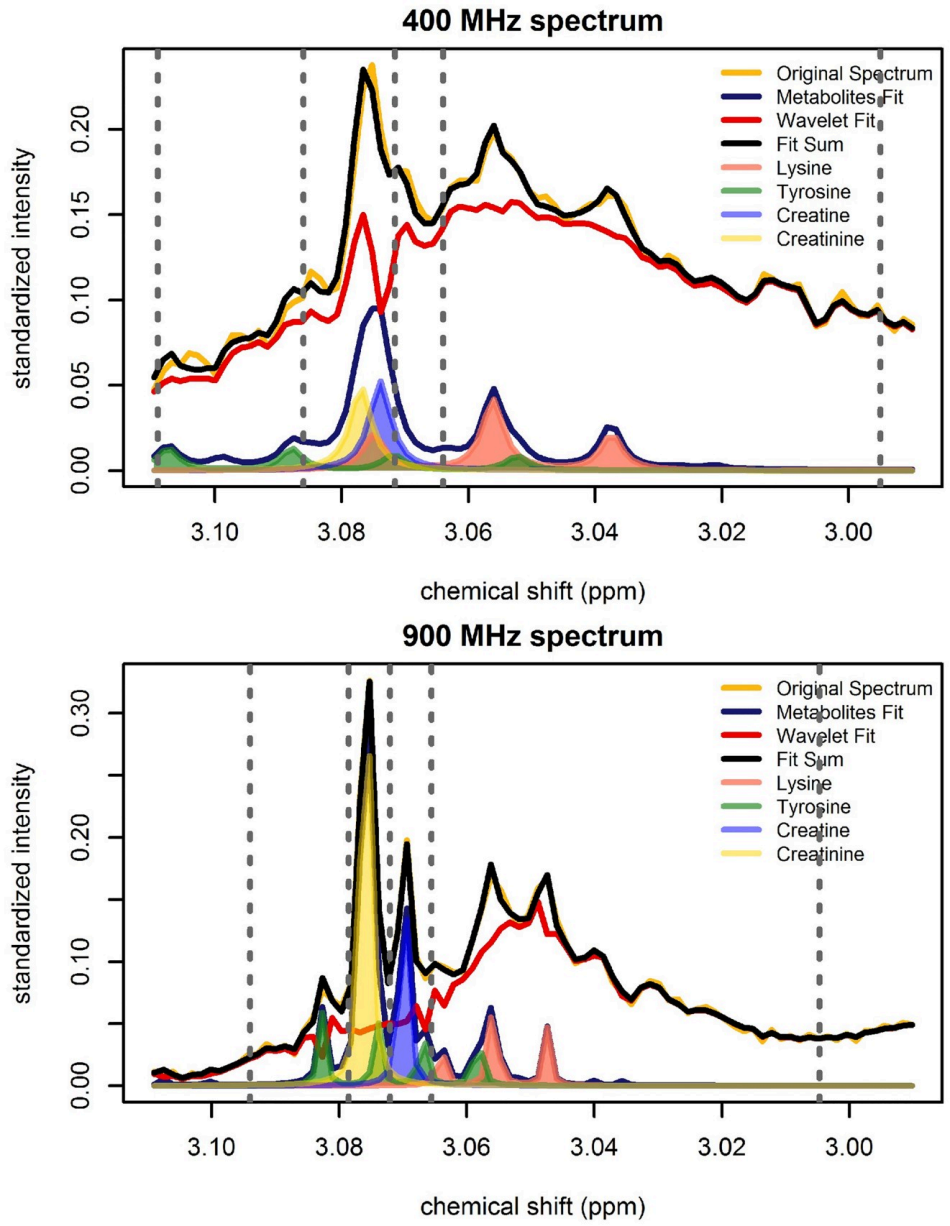
BATMAN fit in the region extending from 2.99 to 3.11 ppm for the 400 MHz spectrum (top) and the 900 MHz spectrum (bottom) of a plasma sample. The original spectrum is shown in yellow. The two components of the BATMAN model fit, that is, the component modeling the metabolic signal (metabolites fit) and the component capturing the residual signal (wavelet fit) are indicated by blue and red curves, respectively. The fit sum which is the sum of the metabolite fit and the wavelet fit is shown in black. The shaded regions show the resonances from creatine (blue), creatinine (yellow), lysine (pink), and tyrosine (green) that are captured by the metabolite fit. The broad lipid = CH–C**H**_2_–CH = resonance in the region is captured by the wavelet fit. Binning integration region limits for the region are delimited by grey dotted lines.

Fig 10 illustrates the fit of the BATMAN model in the region extending from 2.99 to 3.11 ppm for both the 400 MHz and 900 MHz spectrum of a particular plasma sample. This region contains resonances from creatine (singlet), creatinine (singlet), lysine (triplet), and tyrosine (double doublet), as well as a part of the lipid = CH–C**H**_2_–CH = resonance. The resonances are more distinguishable in the 900 MHz spectrum compared to the 400 MHz spectrum. For the 400 MHz spectrum, the four integration regions from left to right aim to capture the signal corresponding to (1) cysteine, lysine, and tyrosine; (2) cysteine, lysine, tyrosine, and creatinine; (3) cysteine, lysine, tyrosine, creatinine, and creatine; and (4) cysteine, lysine, tyrosine, and *α*-ketoglutarate. For the 900 MHz spectrum, the four integration regions from left to right, beginning at 3.0921 ppm, correspond to (1) tyrosine, (2) creatinine, (3) creatine, and (4) lysine and *α*-ketoglutarate.

The top 15 discriminative features, based on the univariate analysis, of each set of predictors are listed in Tables D to H in S1 File.

Comprehensive results of the various classification methods (elastic net, lasso, OPLS-DA, SVMs, and RF) are provided in Figs G to I and Table I in S1 File. Only the elastic net classifiers are further discussed as they proved to be one of the better performing classifiers (see Fig J in S1 File). Table 1 presents the mean cross validated classification error, sensitivity, and specificity of the 400 MHz and 900 MHz classifiers. The 400 MHz classification results indicate that the ISRs (misclassification rate: 0.126, sensitivity: 0.844, specificity: 0.904) had greater predictive power than the relative metabolic and lipid concentrations obtained by using BATMAN (classification error: 0.197, sensitivity: 0.775, specificity: 0.829). For the 900 MHz classification analysis, the relative metabolic concentrations estimated by BATMAN (misclassification rate: 0.105, sensitivity: 0.884, specificity: 0.906) had greater predictive power than the ISRs of the 900 MHz spectral bins (classification error rate: 0.169 (mp), 0.197 (ap); sensitivity: 0.804 (mp), 0.779 (ap); specificity: 0.857 (mp), 0.826 (ap)). Note that for the PepsNMR automatically pre-processed 900 MHz spectra, an additional spectral alignment step was carried out to improve the homogeneity of the bins (in terms of the signal captured) across spectra.

**Table 1:** Elastic net classification results (standard errors in parentheses).

Features	Classification error	Sensitivity	Specificity
400 MHz (manually pre-processed data)			
Binning: top integrated spectral regions^a^	0.126 (0.002)	0.843 (0.003)	0.904 (0.002)
BATMAN: all metabolites and lipids^a^	0.197 (0.002)	0.775 (0.003)	0.829 (0.002)
900 MHz (PepsNMR automatically pre-processed data)			
Binning: top integrated spectral regions^a^	0.197 (0.002)	0.779 (0.003)	0.826 (0.003)
BATMAN: all metabolites^a^	0.105 (0.001)	0.884 (0.002)	0.906 (0.002)
900 MHz (manually pre-processed data)^b^			
Binning: top integrated spectral regions	0.169 (0.002)	0.804 (0.003)	0.857 (0.002)

^a^ Features utilized in Fig 11.

^b^ The 900 MHz manually pre-processed spectra were not of sufficient quality to fit the BATMAN model.

**Fig 11:**
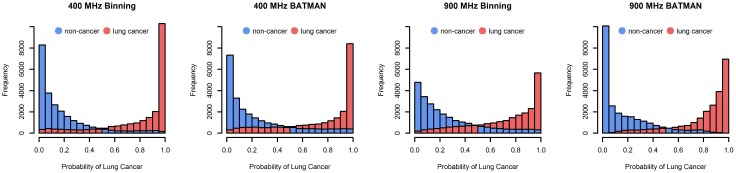
Histograms of the probability of lung cancer based on 333 iterations of three-fold cross-validation. Blue corresponds to the control samples and red represents the lung cancer samples.

Histograms of the probability of lung cancer for the different sets of features are presented in Fig 11. Each histogram is based on the classifiers developed using the subset of features indicated by the letter a in Table 1. Assuming that a probability greater than 0.5 implies the presence of lung cancer, the ISRs of the 400 MHz spectral bins and the 900 MHz relative metabolic concentrations estimated by BATMAN produced the best classifiers in terms of lowest mean classification error and highest sensitivity and specificity.

## 4 Discussion and conclusions

In this study, spectral binning and spectral deconvolution using BATMAN were applied in order to extract metabolic signal from ^1^H-NMR spectra of different spectral resolutions (400 MHz and 900 MHz spectra).

### 4.1 Implementation

Both spectral binning and spectral deconvolution using BATMAN require expert knowledge of the characteristic spectral signatures (i.e., the peak locations and coupling patterns) of different metabolites. For spectral binning, this insight is necessary to select meaningful integration regions. For spectral deconvolution using BATMAN, this information is required to accurately specify and refine the prior information on each multiplet of interest.

Despite BATMAN’s description as an automated metabolite analyzer, an extensive amount of time was spent on developing and fine-tuning the template file in order to improve signal extraction. Although metabolites have characteristic resonances, experimental parameters and pre-processing steps influence the resultant chemical shift positions, identifiable coupling patterns, and relative peak intensities. Note that a single template file is specified for a large number of spectra which exhibit between-spectrum variation in peak shift and peak definition. Thus, template adjustments made to improve the fit of some spectra or peaks may have an opposite effect on others. Updating the template file is a repetitious task which is extremely time-consuming, especially for crowded spectral regions, but it is essential. Once the template database is developed, the process is automated.

Though selecting the integration regions for spectral binning is a manual task, spectral binning is a relatively fast and straightforward method for ^1^H-NMR signal extraction.

The magnetic field strength of the NMR spectrometer influences the resolution of the metabolic peaks. In higher resolution spectra, peaks appear with greater definition, exhibit fewer higher-order effects, and show less overlap. This is beneficial for both spectral binning and spectral deconvolution using the BATMAN model. Fewer overlapping regions imply a greater one-to-one mapping between spectral bins and metabolites [5] and the increased signal-to-noise ratio in the higher resolution spectra is advantageous for metabolic signal extraction using BATMAN (see Fig 10).

### 4.2 Classification and Clinical Relevance

An abundance of detail pertaining to biological functions is contained within the metabolome. There is a strong desire to eventually utilize these data to make informed clinical decisions about disease status, susceptibility, and progression. It is expected that metabolomics will be of vital importance in reaching the goal of providing healthcare that is customized for individual patients. Therefore, obtaining interpretable, reliable, and reproducible results is essential.

The variation in chemical shift locations across spectra is a challenge for spectral binning. Therefore, the inclusion of a spectral alignment step in the pre-processing of NMR data is important in order to obtain reliable and interpretable features. However, even with good spectral alignment, overlapping peaks often prevent a one-to-one mapping between integration regions and metabolites. Integration regions, especially those of lower resolution spectra, may contain signals from two or more metabolites in conjunction with an unidentified signal (for illustration, see Tables D to H in S1 File). Thus, a drawback of the simplicity surrounding spectral binning is the lack of biological interpretability of the resultant features. Nonetheless, for the 400 MHz analysis, the classifier based on the binning features performed better than the one using BATMAN-estimated features.

Spectral deconvolution, particularly the BATMAN model, provides the means to obtain a single concentration estimate for each metabolite of interest. The residual signal captured by wavelets can be divided into integration regions in order to capture for instance, broad lipid resonances. In the end, clinically relevant features are extracted from the ^1^H-NMR spectra. The benefit obtained from the effort put into running BATMAN is biological interpretability. In addition, although not the focus of this manuscript, the reliability of BATMAN estimated relative concentrations can also be assessed by using the 95% credible intervals. For the 900 MHz spectra, the relative metabolic concentrations estimated by BATMAN excelled, producing the best performing classifier in terms of mean misclassification error.

## Supporting information

S1 File Supporting information file. (PDF)

S1 Datasets NMR spectra. (ZIP)
