# Supplementary material for: The impact of the method of extracting metabolic signal from 1H-NMR data on the classification of samples: A case study of binning and BATMAN in lung cancer
Source: PLoS One. 2019 Feb 6;14(2):e0211854. doi: 10.1371/journal.pone.0211854 (PMC6364941; doi:10.1371/journal.pone.0211854)
Supplement: S1 File — (PDF) [file pone.0211854.s001.pdf]

Supporting Information

The impact of the method of extracting metabolic signal from ^{1}H-NMR data on the classification of samples: a case study of binning and BATMAN in lung cancer

Journal: Plos One

Trishanta Padayachee[1]*, Tatsiana Khamiakova[1], Evelyne Louis[2], Peter Adriaensens[3], Tomasz Burzykowski[1]

[1] I-BioStat, Hasselt University, Diepenbeek, Belgium
[2] Faculty of Medicine and Life Sciences, Hasselt University, Diepenbeek, Belgium
[3] Applied and Analytical Chemistry, Institute for Materials Research, Hasselt University, Diepenbeek, Belgium

*Email address of the corresponding author: padayachee.trishanta@gmail.com

Contents

Warping 3
Verifying the goodness of the BATMAN fit 3
Spectral binning regions for the 400 MHz and 900 MHz spectra 4
Parameters of the BATMAN model 10
Regions of the lipid signals 11
BATMAN fit 12
Classification methods 15
Classification results 16
References 24

List of Tables

Table A Spectral binning regions for the 400 MHz and 900 MHz spectra 4
Table B Parameters used to run BATMAN 10
Table C Comparison of the lipid integration regions for the BATMAN and spectral binning analyses 11
Table D Top integration regions for the 400 MHz spectral binning analysis (based on three-fold cross-validation using the limma t-test) 16
Table E Top metabolite/lipid features for the 400 MHz BATMAN analysis (based on three-fold cross-validation using the limma t-test) 16
Table F Top integration regions for the PepsNMR pre-processed 900 MHZ spectral binning analysis (based on three-fold cross-validation using the limma t-test) 17
Table G Top metabolite/lipid features for the PepsNMR pre-processed 900 MHz BATMAN analysis (based on three-fold cross validation using the limma t-test) 17
Table H Top integration regions for the manually pre-processed 900 MHz spectral binning analysis (based on three-fold cross-validation using the limma t-test) 17
Table I Classification results 21

List of Figures

Figure A Illustration of various regions of the warped spectra. 3
Figure B Spectrum 113 showing the resonance of Alanine (blue) together with the signal arising from other metabolites which resonate in the region. 3

Figure C BATMAN mirrored stack plots for a selection of regions in the 400 MHz spectra. Note, the region extending from 1.20 to 1.40 ppm contains a lipid resonance 12
Figure D BATMAN mirrored stack plots for a selection of regions in the 900 MHz spectra. Note, the region extending from 3.00 to 3.12 ppm contains a lipid resonance 13
Figure E Subset of lipid regions extracted from the wavelet component of a 400 MHz spectrum. 14
Figure F Subset of lipid regions extracted from the wavelet component of a 900 MHz spectrum. 14
Figure G Classification performance of the elastic net models utilizing the top k 400 MHz spectral binning integration regions 18
Figure H Classification performance of the elastic net models utilizing the top k PepsNMR pre-processed 900 MHz spectral binning integration regions 19
Figure I Classification performance of the elastic net models utilizing the top k manually pre-processed 900 MHz integration regions 20
Figure J Classification performance in terms of mean misclassification error, mean sensitivity and mean specificity of the elastic net, lasso, orthogonal partial least squares-discriminant analysis (OPLS-DA), random forest (RF), and support vector machine (SVM) 23

Warping

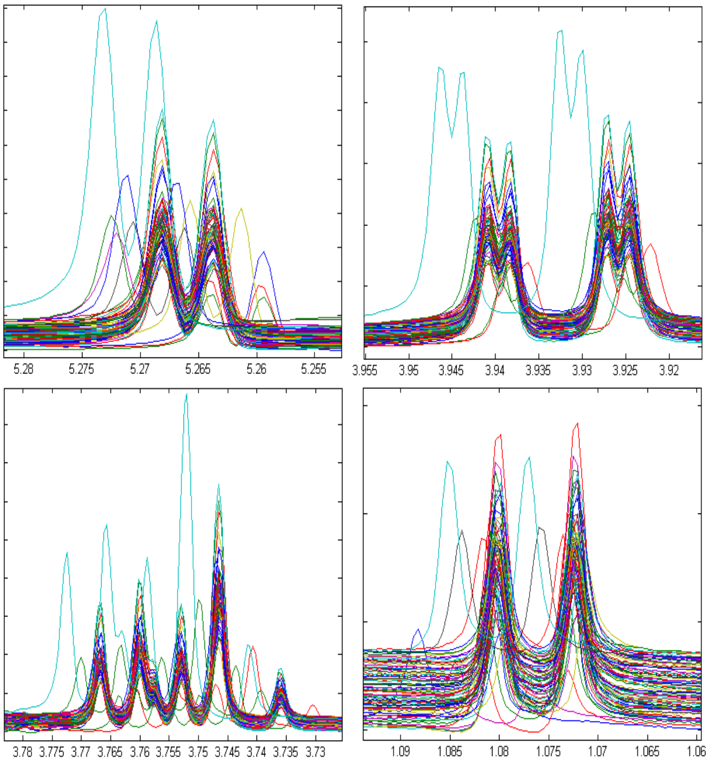

Figure A Illustration of various regions of the warped spectra.

Verifying the goodness of the BATMAN fit

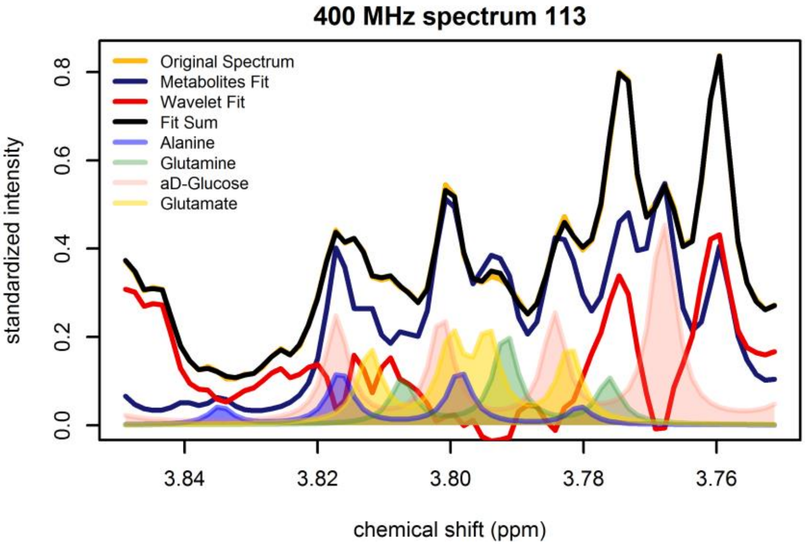

Figure B Spectrum 113 showing the resonance of Alanine (blue) together with the signal arising from other metabolites which resonate in the region.

Spectral binning regions for the 400 MHz and 900 MHz spectra

Table A Spectral binning regions for the 400 MHz and 900 MHz spectra

400 MHz (manually pre-processed spectra)				900 MHz (manually pre-processed spectra)				900 MHz (PepsNMR pre-processed spectra)			
**Region**	**Contributing metabolites**	**Start**	**End**	**Region**	**Contributing metabolites**	**Start**	**End**	**Region**	**Contributing metabolites**	**Start**	**End**
				**1**	NI	8.4914	8.4796	**1**	NI	8.4980	8.4750
				**2**	Formate	8.3702	8.3602	**2**	Formate	8.3810	8.3550
				**3**	NI	8.2601	8.2500	**3**	NI	8.2670	8.2480
				**4**	NI	8.2300	8.2050	**4**	NI	8.2340	8.2000
**1**	NI	7.9500	7.8200	**5**	NI	7.8561	7.8104	**5**	NI	7.8600	7.8200
**2**	His	7.8200	7.7890								
**3**	NI	7.7890	7.7780	**6**	His	7.7812	7.7544	**6**	His	7.8000	7.7644
**4**	His	7.7780	7.7480								
**5**	NI	7.7480	7.7200								
**6**	NI	7.6800	7.5920								
**7**	NI	7.5920	7.5480								
**8**	Phe	7.4840	7.3620	**7**	Phe	7.4677	7.4380	**7**	Phe	7.4750	7.4380
				**8**	Phe, NI	7.4162	7.3755	**8**	Phe, NI	7.4210	7.3755
**9**	Phe, NI	7.3620	7.3300	**9**	Phe	7.3675	7.3484	**9**	Phe	7.3750	7.3510
**10**	NI	7.3300	7.2820	**10**	NI	7.3484	7.3227	**10**	NI	7.3510	7.3227
**11**	NI	7.2820	7.2550								
**12**	Tyr, NI	7.2550	7.2390								
**13**	Tyr, NI	7.2390	7.2000	**11**	Tyr	7.2327	7.2046	**11**	Tyr	7.2400	7.2046
				**12**	NI	7.1894	7.1591	**12**	NI	7.1894	7.1591
**14**	His	7.1070	7.0656	**13**	His	7.0792	7.0597	**13**	His	7.0880	7.0600
				**14**	NI	7.0201	6.9652	**14**	NI	7.0201	6.9600
**15**	Tyr	6.9430	6.9050	**15**	Tyr	6.9355	6.9056	**15**	Tyr	6.9440	6.9056
**16**	NI	6.9050	6.8810								

400 MHz (manually pre-processed spectra)				900 MHz (manually pre-processed spectra)				900 MHz (PepsNMR pre-processed spectra)			
**Region**	**Contributing metabolites**	**Start**	**End**	**Region**	**Contributing metabolites**	**Start**	**End**	**Region**	**Contributing metabolites**	**Start**	**End**
**17**	NI	6.7445	6.7020	**16**	NI	6.7460	6.7004	**16**	NI	6.7600	6.7004
**18**	Lipids:–C**H**=C**H**– in FAC	5.4300	5.2752	**17**	Lipids:–C**H**=C**H**– in FAC	5.4422	5.2833	**17**	Lipids:–C**H**=C**H**– in FAC	5.4422	5.2900
**19**	Glucose	5.2752	5.2526	**18**	Glucose	5.2751	5.2542	**18**	Glucose	5.2770	5.2560
**20**	C_2H in glycerol backbone of PL and TG	5.2526	5.2030	**19**	C_2H in glycerol backbone of PL and TG	5.2542	5.2301	**19**	C_2H glycerol backbone of PL and TG	5.2560	5.2301
				**20**		5.2186	5.2038	**20**		5.2260	5.2038
				**21**	NI	5.1525	5.1187	**21**	NI	5.1550	5.1187
**21**	Glucose	4.6940	4.6620	**22**	Glucose	4.7088	4.6421	**22**	Glucose	4.7088	4.6421
**22**	NI	4.5560	4.5380								
**23**	NI	4.5380	4.4100								
**24**	C_1H and C_3H in glycerol backbone of TG	4.4100	4.3159	**23**	C_1H and C_3H in glycerol backbone of TG	4.3579	4.2902	**23**	C_1H and C_3H in glycerol backbone of TG	4.3579	4.2940
**25**	O–C$\mathbf{H}_2$–CH_2–$N^+(CH_3)_3$ of PC and SM, Thr	4.3159	4.2332	**24**	O–C$\mathbf{H}_2$–CH_2–$N^+(CH_3)_3$ of PC and SM, Thr	4.2852	4.2536	**24**	O–C$\mathbf{H}_2$–CH_2–$N^+(CH_3)_3$ of PC and SM, Thr	4.2940	4.2500
**26**	β-hydroxybutyrate, Pro	4.2000	4.1885	**25**	β-hydroxybutyrate	4.2000	4.1607	**25**	β-hydroxybutyrate	4.2000	4.1607
**27**	β-hydroxybutyrate, Pro, Lactate	4.1885	4.1750								
**28**	C_1H and C_3H glycerol backbone of PL and TG, lactate	4.1750	4.1260	**26**	C_1H and C_3H glycerol backbone of PL and TG, lactate	4.1570	4.1276	**26**	C_1H and C_3H glycerol backbone of PL and TG, lactate	4.1600	4.1276
**29**	NI	4.1260	4.1110	**27**	NI	4.1276	4.0942	**27**	NI	4.1276	4.0950
**30**	NI	4.1110	4.1032								
**31**	Creatinine	4.1032	4.0700	**28**	Creatinine	4.0904	4.0780	**28**	Creatinine	4.0950	4.0770
**32**	NI	4.0700	4.0570								
**33**	His, Ser	4.0570	4.0310								
**34**	Asn, His, Phe, Ser	4.0310	4.0136	**29**	C_3H_2 in glycerol backbone of PL, Asn, His, Phe, Ser	4.0400	3.9913	**29**	C_3H_2 in glycerol backbone of PL, Asn, His, Phe, Ser	4.0420	3.9920
**35**	C_3H_2 in glycerol backbone of PL, Asn, His, Phe, Ser	4.0136	4.0010								
**36**	C_3H_2 in glycerol backbone of PL, Asn, His, Phe, Ser	4.0010	3.9810								
**37**	Creatine, Asn, His, Tyr, Ser	3.9810	3.9590	**30**	Asn, His, Ser, Tyr	3.9903	3.9644	**30**	Asn, His, Ser, Tyr	3.9920	3.9680
				**31**	Creatine	3.9644	3.9586	**31**	Creatine	3.9680	3.9600
				**32**	Tyr	3.9586	3.9527	**32**	Tyr	3.9600	3.9527

400 MHz (manually pre-processed spectra)			
**Region**	**Contributing metabolites**	**Start**	**End**
**38**	Glucose, Asp, Met, Ser, Tyr	3.9590	3.8330
**39**	Glucose, Ala, Ser	3.8330	3.8100
**40**	Glucose, Ala, Gln, Glu	3.8100	3.7956
**41**	Glucose, Ala, Gln, Glu, Leu, Lys	3.7956	3.7820
**42**	Glucose, Ala, Gln, Glu, Leu, Lys	3.7820	3.7550
**43**	Glucose, Ala, Leu	3.7550	3.7390
**44**	Glucose	3.7390	3.7141
**45**	$O{-}CH_2{-}C\mathbf{H}_2{-}N^+(CH_3)_3$ of PC and SM, glycerol, Ile	3.7141	3.6680
**46**	Glycerol	3.6680	3.6500
**47**	Glycerol, Val	3.6500	3.6376
**48**	Val	3.6376	3.6240
**49**	Thr	3.6240	3.6097
**50**	Thr	3.6097	3.5914
**51**	Glucose, glycerol, Gly, Thr	3.5914	3.5649
**52**	Glucose	3.5649	3.5510
**53**	Glucose, acetoacetate, Pro	3.5510	3.5360
**54**	Glucose, acetoacetate, Pro	3.5360	3.3980
**55**	Methanol, NI	3.3980	3.3765
**56**	Pro	3.3765	3.3430
**57**	Phe, Pro	3.3430	3.3230

900 MHz (manually pre-processed spectra)			
**Region**	**Contributing metabolites**	**Start**	**End**
**33**	Glucose	3.9527	3.9120
**34**	Glucose	3.9120	3.8957
**35**	Glucose	3.8881	3.8306
**36**	Glucose, Ala, Gln, Glu, Ser	3.8286	3.8097
**37**	Glucose, Ala, Gln	3.8097	3.7794
**38**	Glucose	3.7776	3.7275
**39**	Glycerol	3.7204	3.6453
**40**	Val	3.6453	3.6212
**41**	Thr	3.6163	3.5861
**42**	Glycerol	3.5861	3.5771
**43**	Glucose	3.5771	3.5481
**44**	Glucose	3.5355	3.4798
**45**	Pro	3.4772	3.4576
**46**	Glucose	3.4576	3.4093
**47**	Methanol	3.3964	3.3924
**48**	NI	3.3924	3.3746
**49**	Pro	3.3746	3.3465

900 MHz (PepsNMR pre-processed spectra)			
**Region**	**Contributing metabolites**	**Start**	**End**
**33**	Glucose	3.9527	3.9150
**34**	Glucose	3.9150	3.8920
**35**	Glucose	3.8920	3.8410
**36**	Glucose, Ala, Gln, Glu, Ser	3.8410	3.8140
**37**	Glucose, Ala, Gln	3.8140	3.7794
**38**	Glucose	3.7776	3.7275
**39**	Glycerol	3.7240	3.6500
**40**	Val	3.6500	3.6250
**41**	Thr	3.6163	3.5930
**42**	Glycerol	3.5930	3.5810
**43**	Glucose	3.5810	3.5481
**44**	Glucose	3.5481	3.4798
**45**	Pro	3.4798	3.4600
**46**	Glucose	3.4600	3.4093
**47**	Methanol	3.4004	3.3924
**48**	NI	3.3924	3.3770
**49**	Pro	3.3770	3.3465

400 MHz (manually pre-processed spectra)			
**Region**	**Contributing metabolites**	**Start**	**End**
**58**	$O–CH_2–CH_2–N^+(C\mathbf{H}_3)_3$ of PC and SM, glucose, His, Phe, Tyr	3.3230	3.2186
**59**	Tyr, NI	3.2186	3.1930
**60**	NI	3.1930	3.1760
**61**	NI	3.1760	3.1462
**62**	His, Phe	3.1462	3.1090
**63**	Lys, Tyr	3.1090	3.0860
**64**	Creatinine, Lys, Tyr	3.0860	3.0716
**65**	Creatinine, creatine, Lys	3.0716	3.0640
**66**	α-ketoglutarate, Lys	3.0640	2.9950
**67**	Lipids: $=CH–C\mathbf{H}_2–CH=$ in FAC, Asn	2.9950	2.8860
**68**	Lipids: $=CH–C\mathbf{H}_2–CH=$ in FAC, Asn, Asp	2.8860	2.8550
**69**	Lipids: $=CH–C\mathbf{H}_2–CH=$ in FAC, Asn, Asp	2.8550	2.7500
**70**	Citrate, Asp	2.7500	2.7360
**71**	Citrate, Asp, Met	2.7360	2.6600
**72**	Met	2.6600	2.6300
**73**	Citrate	2.5960	2.5340
**74**	NI	2.5340	2.5150

900 MHz (manually pre-processed spectra)			
**Region**	**Contributing metabolites**	**Start**	**End**
**50**	Phe	3.3256	3.3132
**51**	Phe, NI	3.3132	3.3030
**52**	NI	3.3030	3.2956
**53**	NI	3.2956	3.2909
**54**	Glucose	3.2909	3.2616
**55**	$O–CH_2–CH_2–N^+(C\mathbf{H}_3)_3$ of PC and SM	3.2616	3.2085
**56**	Tyr, NI	3.1972	3.1895
**57**	NI	3.1881	3.1821
**58**	NI	3.1821	3.1724
**59**	NI	3.1707	3.1571
**60**	His, Phe	3.1541	3.1378
**61**	Tyr	3.0921	3.0769
**62**	Creatinine	3.0769	3.0699
**63**	Creatine	3.0699	3.0635
**64**	α-ketoglutrate, Lys	3.0635	3.0047
**65**	Lipids: $=CH–C\mathbf{H}_2–CH=$ in FAC	3.0047	2.9655
**66**	Asn	2.9597	2.9201
**67**	Lipids: $=CH–C\mathbf{H}_2–CH=$ in FAC, Asn, Asp	2.8874	2.8465
**68**	Lipids: $=CH–C\mathbf{H}_2–CH=$ in FAC	2.8465	2.7623
**69**	Citrate	2.7571	2.7493
**70**	NI	2.7472	2.7390
**71**	Citrate	2.7368	2.7251
**72**	Asp	2.7237	2.6768
**73**	Met	2.6768	2.6597
**74**	Citrate	2.5865	2.5426

900 MHz (PepsNMR pre-processed spectra)			
**Region**	**Contributing metabolites**	**Start**	**End**
**50**	Phe	3.3300	3.3160
**51**	Phe, NI	3.3160	3.3050
**52**	NI	3.3050	3.2950
**53**	NI	3.2950	3.2920
**54**	Glucose	3.2920	3.2646
**55**	$O–CH_2–CH_2–N^+(C\mathbf{H}_3)_3$ of PC and SM	3.2640	3.2085
**56**	Tyr, NI	3.2000	3.1900
**57**	NI	3.1900	3.1800
**58**	NI	3.1800	3.1724
**59**	NI	3.1724	3.1600
**60**	His, Phe	3.1600	3.1300
**61**	Tyr	3.0940	3.0785
**62**	Creatinine	3.0785	3.0720
**63**	Creatine	3.0720	3.0655
**64**	α-ketoglutrate, Lys	3.0655	3.0047
**65**	Lipids: $=CH–C\mathbf{H}_2–CH=$ in FAC	3.0047	2.9655
**66**	Asn	2.9655	2.9201
**67**	Lipids: $=CH–C\mathbf{H}_2–CH=$ in FAC, Asn, Asp	2.8874	2.8450
**68**	Lipids: $=CH–C\mathbf{H}_2–CH=$ in FAC	2.8465	2.7623
**69**	Citrate	2.7530	2.7250
**70**	Asp	2.7250	2.6900
**71**	Met	2.6900	2.6597
**72**	Citrate	2.5865	2.5426

400 MHz (manually pre-processed spectra)				900 MHz (manually pre-processed spectra)				900 MHz (PepsNMR pre-processed spectra)			
**Region**	**Contributing metabolites**	**Start**	**End**	**Region**	**Contributing metabolites**	**Start**	**End**	**Region**	**Contributing metabolites**	**Start**	**End**
**75**	Gln	2.5150	2.4920	**75**	Gln	2.5183	2.4428	**73**	Gln	2.5250	2.4440
**76**	β-hydroxybutyrate, α-ketoglutarate, Gln	2.4920	2.4500								
**77**	β-hydroxybutyrate, α-ketoglutarate, succinate	2.4500	2.4324	**76**	β-hydroxybutyrate	2.4428	2.4280	**74**	β-hydroxybutyrate	2.4440	2.4280
**78**	β-hydroxybutyrate, Pro	2.4324	2.4148								
**79**	β-hydroxybutyrate, Glu, Pro	2.4148	2.4050								
**80**	Pyruvate, Pro, Glu	2.4050	2.3990	**77**	Pyruvate	2.4060	2.3978	**75**	Pyruvate	2.4110	2.3980
**81**	β-hydroxybutyrate, Pro, Glu	2.3990	2.3640	**78**	Glu	2.3978	2.3648	**76**	Glu	2.4000	2.3600
**82**	β-hydroxybutyrate, Pro, Glu	2.3640	2.3500								
**83**	β-hydroxybutyrate, Pro, Val	2.3500	2.3380	**79**	β-hydroxybutyrate	2.3540	2.3194	**77**	β-hydroxybutyrate	2.3540	2.3194
**84**	β-hydroxybutyrate , acetoacetate, Pro, Val	2.3380	2.3170								
**85**	β-hydroxybutyrate, acetoacetate, Val	2.3170	2.3040	**80**	Acetoacetate	2.3134	2.3067	**78**	Acetoacetate	2.3194	2.3055
**86**	Lipids: –C**H**$_2$–C=O or –C**H**$_2$–CH=CH– in FAC, Val, β-hydroxybutyrate	2.3040	2.2915	**81**	Lipids: –C**H**$_2$–C=O or –C**H**$_2$–CH=CH– in FAC	2.3067	2.2630	**79**	Lipids: –C**H**$_2$–C=O or –C**H**$_2$–CH=CH– in FAC	2.2990	2.2680
**87**	Lipids: –C**H**$_2$–C=O or –C**H**$_2$–CH=CH– in FAC, Met, Val	2.2915	2.2690								
**88**	Lipids: –C**H**$_2$–C=O or –C**H**$_2$–CH=CH– in FAC, acetone, Met, Val	2.2690	2.2300	**82**	Acetone	2.2630	2.2563	**80**	Acetone	2.2680	2.2563
**89**	Glu, Met	2.2180	2.1970								
**90**	Gln, Glu, Pro, Met	2.1970	2.1230	**83**	NI	2.1975	2.1814	**81**	NI	2.1975	2.1930
				**84**	Gln	2.1777	2.1670	**82**	Gln	2.1930	2.1700
				**85**	Met	2.1670	2.1919	**83**	Met	2.1700	2.1650
				**86**	Gln	2.1619	2.1311	**84**	Gln	2.1650	2.1311
**91**	Lipids: –C**H**$_2$–CH=CH– in FAC, C**H**$_3$ of NAG, Glu, Ile, Met, Pro	2.1230	1.9720	**87**	Lipids: –C**H**$_2$–CH=CH– in FAC	2.1289	2.0993	**85**	Lipids: –C**H**$_2$–CH=CH– in FAC	2.1300	2.0975
				**88**	Lipids: –C**H**$_2$–CH=CH– in FAC, C**H**$_3$ of NAG	2.0993	1.9889	**86**	Lipids: –C**H**$_2$–CH=CH– in FAC, C**H**$_3$ of NAG	2.0985	1.9889
**92**	Acetate, Ile, Lys	1.9720	1.9240								
				**89**	Acetate	1.9547	1.9421	**87**	Acetate	1.9547	1.9450

400 MHz (manually pre-processed spectra)				900 MHz (manually pre-processed spectra)				900 MHz (PepsNMR pre-processed spectra)			
**Region**	**Contributing metabolites**	**Start**	**End**	**Region**	**Contributing metabolites**	**Start**	**End**	**Region**	**Contributing metabolites**	**Start**	**End**
**92**				**90**	Lys	1.9421	1.9028	**88**	Lys	1.9450	1.9100
**93**	Ile, Lys	1.9240	1.8800								
**94**	Leu, Lys	1.8060	1.6860	**91**	Leu	1.8006	1.6758	**89**	Leu	1.8006	1.6758
**95**	Lipids: –C**H**$_2$–CH$_2$–C=O or –C**H**$_2$–CH$_2$–CH=CH– in FAC, Lys	1.6860	1.5600	**92**	Lipids: –C**H**$_2$–CH$_2$–C=O or –C**H**$_2$–CH$_2$–CH=CH– in FAC	1.6530	1.5770	**90**	Lipids: –C**H**$_2$–CH$_2$–C=O or –C**H**$_2$–CH$_2$–CH=CH– in FAC	1.6530	1.5770
**96**	Ala, Ile, Lys	1.5400	1.4900	**93**	Ala	1.5226	1.4919	**91**	Ala	1.5226	1.4850
**97**	Ile, Leu, Lys	1.4900	1.4200	**94**	Lys	1.4587	1.4201	**92**	Lys	1.4587	1.4201
**98**	Lactate	1.4200	1.3740	**95**	Lactate	1.4169	1.3675	**93**	Lactate	1.4169	1.3730
**99**	Lactate, Thr	1.3740	1.3450	**96**	Lactate	1.3675	1.3516	**94**	Lactate	1.3730	1.3516
**100**	Lipids: –CH$_3$–(C**H**$_2$)$_n$- FAC, Ile, Thr	1.3450	1.2458	**97**	Lipids: –CH$_3$–(C**H**$_2$)$_n$- FAC	1.3516	1.2366	**95**	Lipids: –CH$_3$–(C**H**$_2$)$_n$- FAC	1.3500	1.2500
**101**	β-hydroxybutyrate, Ile	1.2458	1.2180	**98**	β-hydroxybutyrate	1.2366	1.2240	**96**	β-hydroxybutyrate	1.2500	1.2250
**102**	NI	1.2180	1.1300	**99**	NI	1.2240	1.1766	**97**	NI	1.2200	1.1700
**103**	Val	1.0930	1.0610	**100**	Val	1.0860	1.0592	**98**	Val	1.0950	1.0620
**104**	Ile	1.0610	1.0400	**101**	Ile	1.0513	1.0340	**99**	Ile	1.0620	1.0370
**105**	Ile, Val	1.0400	1.0220	**102**	Val	1.0396	1.0106	**100**	Val	1.0370	1.0150
**106**	Ile, Leu, Val	1.0220	1.0020								
**107**	Ile, Leu	1.0020	0.9860	**103**	Leu	1.0083	0.9766	**101**	Leu	1.0150	0.9800
**108**	Ile, Leu	0.9860	0.9760								
**109**	Ile	0.9760	0.9660	**104**	Ile	0.9766	0.9663	**102**	Ile	0.9800	0.9550
**110**	Lipids: C**H**$_3$–(CH$_2$)$_n$– in FAC	0.9660	0.8000	**105**	Lipids: C**H**$_3$–(CH$_2$)$_n$– in FAC	0.9663	0.7961	**103**	Lipids: C**H**$_3$–(CH$_2$)$_n$– in FAC	0.9663	0.7961

Abbreviations: NI: not identified, FAC: fatty acid chain, NAG: N-acetylated glycoproteins, PC: phosphatidylcholine, PL: phospholipids, SM: sphingomyelins, TG: triglycerides

Ala: Alanine, Arg: Arginine, Asn: Asparagine, Asp: Aspartate, Cys: Cysteine, Gln: Glutamine, Glu: Glutamate, Gly: Glycine, His: Histidine, Ile: Isoleucine, Leu: Leucine, Lys: Lysine, Met: Methionine, Phe: Phenylalanine, Pro: Proline, Ser: Serine, Thr: Threonine, Trp: Tryptophan, Tyr: Tyrosine, Val: Valine

Parameters of the BATMAN model

The parameters used to fit the BATMAN model were selected based on the properties of the spectra and the recommendations of Hao et al. (2014)[1] (see Table S2). The truncation threshold for negative intensities was lower for the 400 MHz analysis compared to the 900 MHz analysis in order to accommodate the negative intensities (due to minor phasing issues) in some of the 400 MHz spectra. As per the recommendations of Hao et al. (2014)[1], the parameters controlling the wavelet fit (i.e., shape (a) and scale (b) in Table S2) were left at their default values. Peaks were allowed to shift more in the 400 MHz analysis as greater variation was observed in the location of the multiplets across the 400 MHz spectra.

Table B Parameters used to run BATMAN

BATMAN options file parameters	400 MHz spectra	900 MHz spectra
General parameters		
Truncation threshold for negative intensities	-0.5	-0.05
Intensity scale factor	100	100
Down sampling factor	3	3
Number of burn-in iterations	3500	3500
Number of post-burn-in iterations	1500	1500
Spectrometer frequency (MHz)	399.793	900.2298630
Uncatalogued (wavelet) component		
Shape (a)	0.00001	0.00001
Scale (b)	0.000000001	0.000000001
Catalogued metabolite component		
Mean of prior on global peak width (μ) in ln(Hz)	0	0
Variance of prior on global peak width (μ) in ln(Hz)	0.01	0.01
Variance of proposal distribution for μ in ln(Hz)	0.002	0.002
Variance of prior on peak width offset (ν_m) in ln(Hz)	0.0025	0.0025
Variance of proposal distribution for ν_m in ln(Hz)	0.1	0.1
Wavelet truncation		
Mean of the prior on τ	-0.05	-0.05
Inverse of variance of prior on τ	2	1
Peak shift		
Truncation of prior on peak shift (ppm)	0.01	0.005

Regions of the lipid signals

Table C Comparison of the lipid integration regions for the BATMAN and spectral binning analyses

Lipid features	Manually pre-processed 400 MHz spectra Spectral binning♦		Manually pre-processed 400 MHz spectra BATMAN		PepsNMR pre-processed 900 MHz spectra Spectral binning		PepsNMR pre-processed 900 MHz spectra BATMAN	
	Start	End	Start	End	Start	End	Start	End
–C**H**=C**H**– in FAC	5.4300	5.2752	5.4300	5.2800	5.4422	5.2900	5.4200	5.2833
albumin (lysyl)			3.3500	3.1500			3.2700	3.1500
=CH–C$\mathbf{H_2}$–CH= in FAC (b)	2.9950<br>2.8860	2.8860<br>2.8550	3.1000	2.9000	3.0047<br>2.8874	2.9655<br>2.8465	3.1000	2.9000
=CH–C$\mathbf{H_2}$–CH= in FAC (a)	2.8550	2.7500	2.8880	2.6500	2.8465	2.7623	2.8880	2.6500
–C$\mathbf{H_2}$–C=O<br>or<br>–C$\mathbf{H_2}$–CH=CH– in FAC	2.3040<br>2.2915<br>2.2690	2.2915<br>2.2690<br>2.2300	2.3060	2.2300	2.2990	2.2680	2.3060	2.2630
–C$\mathbf{H_2}$–CH=CH– in FAC and C$\mathbf{H_3}$ in NAG	2.1230	1.9720	2.1500	1.9500	2.1300<br>2.0985	2.0975<br>1.9889	2.1289	1.9889
–C$\mathbf{H_2}$–CH_2–C=O<br>or<br>–C$\mathbf{H_2}$–CH_2–CH=CH– in FAC	1.6860	1.5600	1.6860	1.5400	1.6530	1.5770	1.6530	1.5770
–CH_3–(C$\mathbf{H_2}$)$_n$– in FAC	1.3450	1.2458	1.4300	1.1600	1.4169<br>1.3516	1.3730<br>1.2500	1.4169<br>1.3516	1.3675<br>1.2366
C$\mathbf{H_3}$–(CH_2)$_n$– in FAC	0.9660	0.8000	0.9660	0.8000	0.9663	0.7961	0.9660	0.7961

Abbreviations: FAC: fatty acid chain, NAG: N-acetylated glycoproteins

♦ As reported in Louis et al. (2015)[2].

BATMAN fit

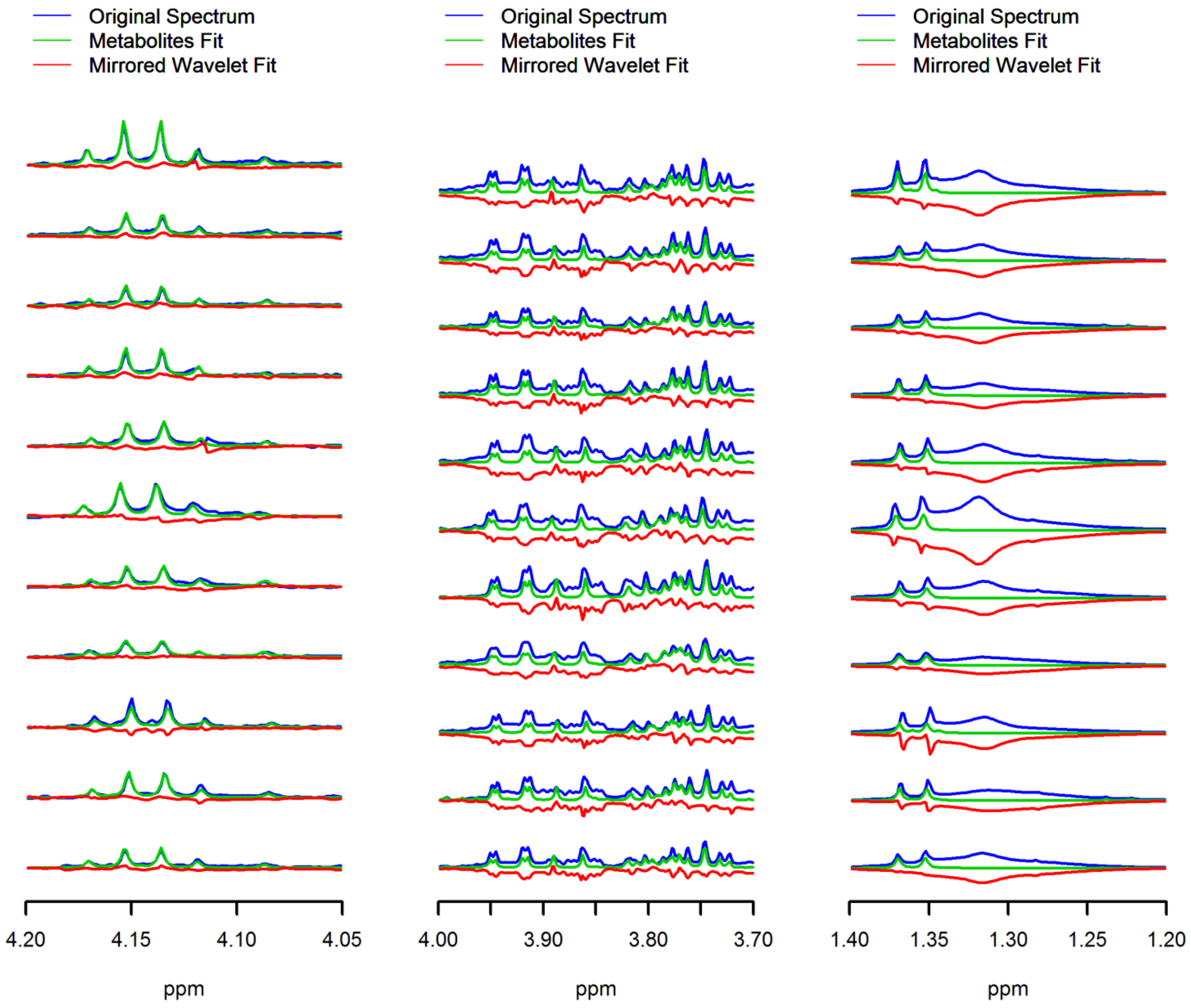

Figure C BATMAN mirrored stack plots for a selection of regions in the 400 MHz spectra. Note, the region extending from 1.20 to 1.40 ppm contains a lipid resonance.

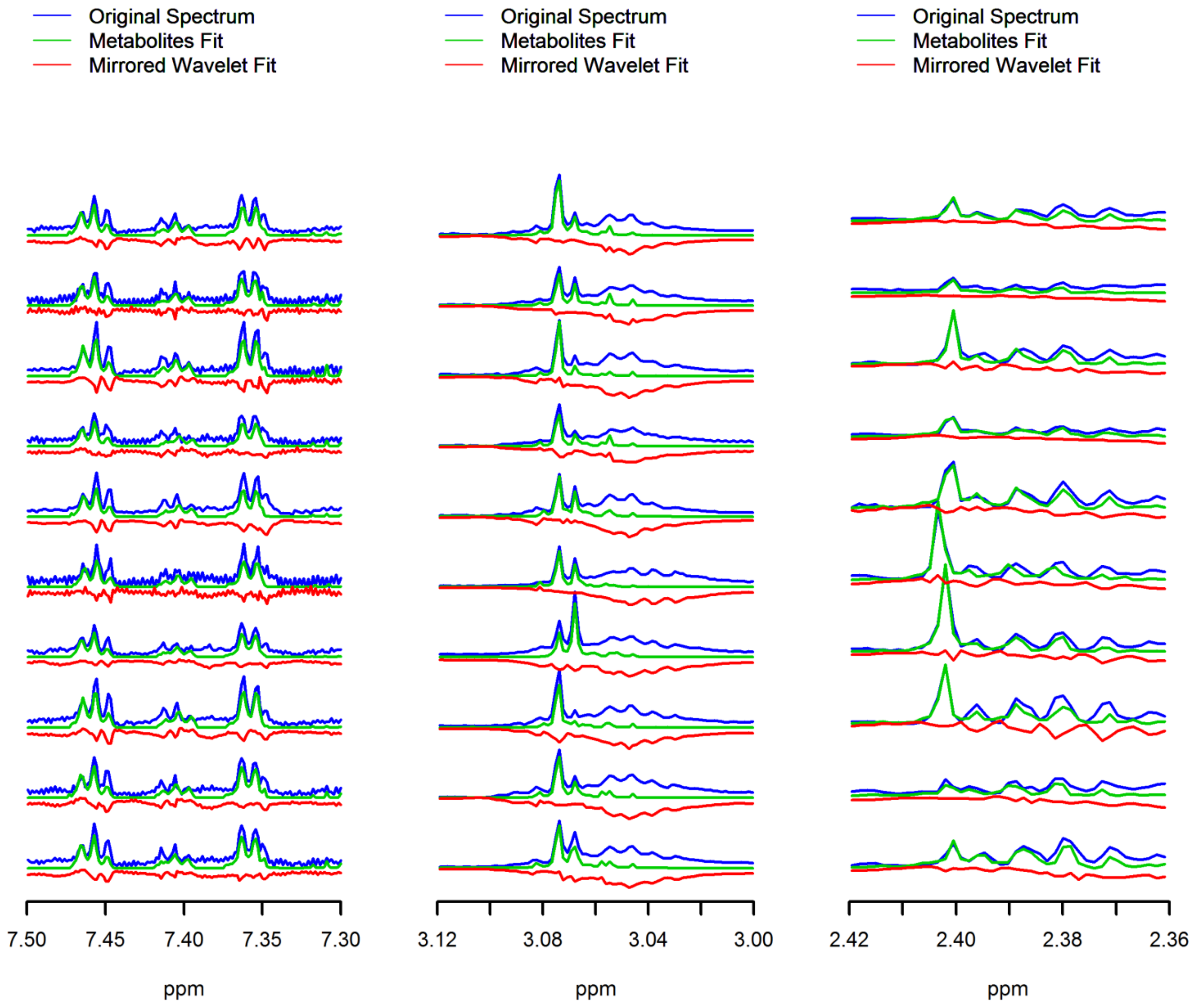

Figure D BATMAN mirrored stack plots for a selection of regions in the 900 MHz spectra. Note, the region extending from 3.00 to 3.12 ppm contains a lipid resonance.

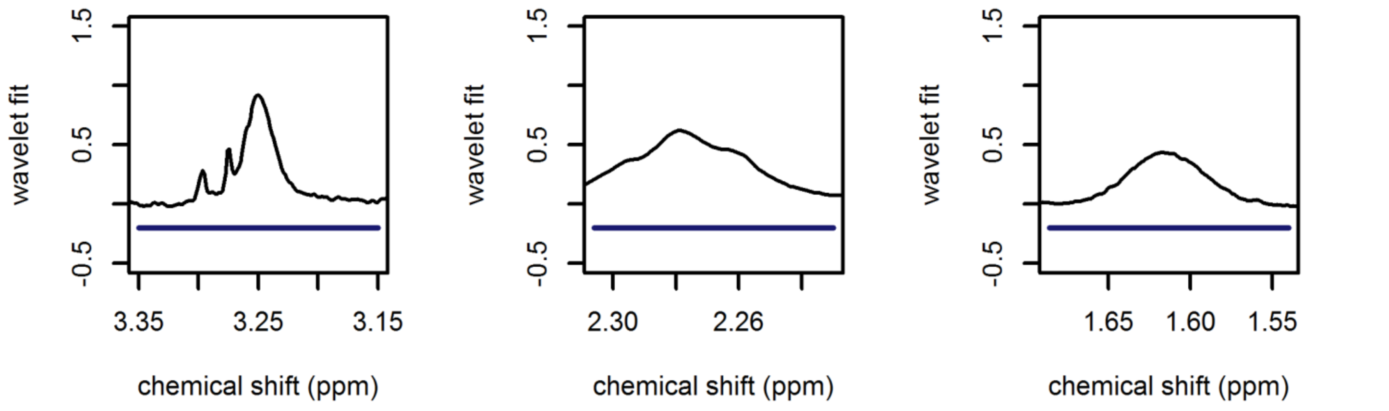

Figure E Subset of lipid regions extracted from the wavelet component of a 400 MHz spectrum.

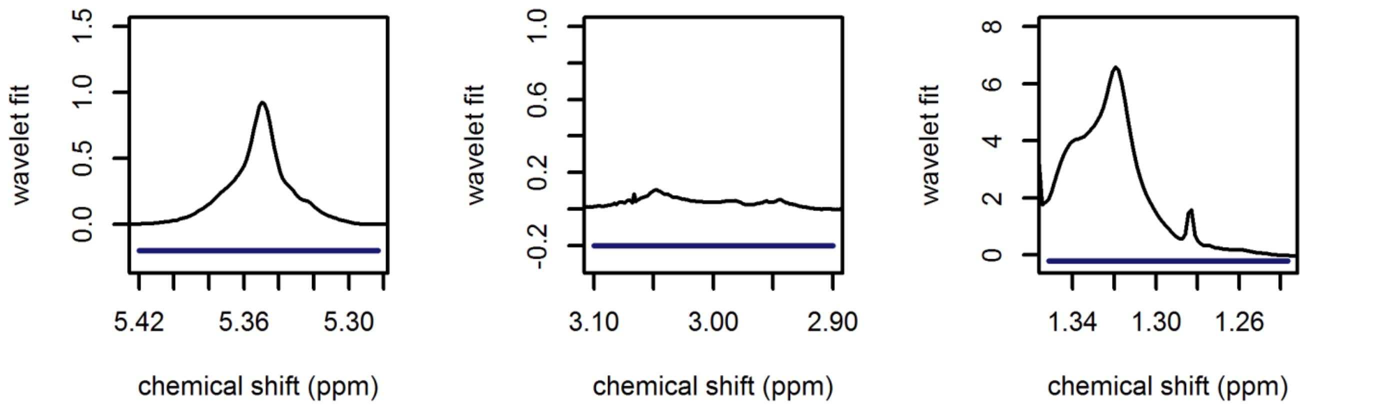

Figure F Subset of lipid regions extracted from the wavelet component of a 900 MHz spectrum.

Classification methods

A brief description of each of the classification methods used is provided here, and the reader is referred to Hastie et al. (2009)[3] and Bylesjö et al., (2006)[4] for further details.

Lasso and elastic net are both regularized regression procedures (i.e., penalty terms are added to the regression framework which is logistic regression in our case). Lasso utilizes the L1 penalty which constrains the sum of absolute values of the regression coefficients. Lasso enables variable selection as L1 regularization allows for some regression coefficients to be shrunk to zero. Ridge regression, also a regularized regression procedure, utilizes the L2 penalty which constrains the sum of squared regression coefficients. Regularization with the L2 penalty is good for handling correlated predictors. Elastic net combines the L1 penalty of lasso with the L2 penalty of ridge regression. Thus, elastic net enables variable selection and allows for strongly correlated predictors to either enter or be left out of the model together.

Partial least squares-discriminant analysis (PLS-DA) and orthogonal partial least squares-discriminant analysis (OPLS-DA) are well-known multivariate-regression approaches, used for performing classification, in metabolomics. Given two matrices, a response matrix Y containing class information and a matrix of predictors X, PLS-DA models X and Y simultaneously with the aim of maximizing the covariance between X and Y. The procedure identifies latent variables (a.k.a. PLS components) in X that are predictive of Y. In OPLS-DA, the information contained in Y is used to split the X matrix into blocks that are correlated with Y, capturing the predictive variation, and orthogonal to Y, capturing the non-predictive variation. Thus, the variation in X that is not correlated with Y is removed. This reduces the complexity of the final model making the results more interpretable.

SVMs search for a hyperplane that maximizes the margin of separation between the two classes. RF classifiers combine a number of decision trees each based on bootstrapped samples of the training dataset.

Classification results

Univariate analysis

Limma was applied in three-fold cross-validation as a univariate approach to identify the top 15 variables of each of the five sets of predictors. For each training dataset in the three-fold cross-validation procedure, the predictors are ranked according to their associated limma t-test statistics. The top ranking k = 15 features of each dataset are selected. The frequency with which each feature appears in the collection of top 15 feature lists is computed and those features appearing most frequently in the top 15 lists across the iterations appear in Tables S4 to S8.

Table D Top integration regions for the 400 MHz spectral binning analysis (based on three-fold cross-validation using the limma t-test)

Top 15 integration regions	
Integration region	Signal found in region
[4.0010, 3.9810]	C_3H_2 in glycerol backbone of PL, Asparagine, Histidine, Phenylalanine, Serine
[3.9810, 3.9590]	Creatine, Asparagine, Histidine, Tyrosine, Serine
[3.6376, 3.6240]	Valine
[4.1260, 4.1110]	Not identified
[3.6240, 3.6097]	Threonine
[2.1230, 1.9720]	Lipids: –C**H**$_2$–CH=CH– in FAC, C**H**$_3$ of NAG, Glutamate, Isoleucine, Methionine, Proline
[3.7141, 3.6680]	O-CH$_2$-C**H**$_2$-N$^+$(CH$_3$)$_3$ of PC and SM, Glycerol, Isoleucine
[4.1750, 4.1260]	C_1H and C_3H in glycerol backbone of PL and TG, Lactate
[3.6680, 3.6500]	Glycerol
[4.5380, 4.4100]	Not identified
[3.6097, 3.5914]	Threonine
[0.9760, 0.9660]	Isoleucine
[7.8200, 7.7890]	Histidine
[2.5960, 2.5340]	Citrate
[3.9590, 3.8330]	Glucose, Aspartate, Methionine, Serine, Tyrosine

Abbreviations: FAC: fatty acid chain, NAG: N-acetylated glycoproteins, PC: phosphatidylcholine, PL: phospholipids, SM: sphingomyelins, TG: triglycerides

Table E Top metabolite/lipid features for the 400 MHz BATMAN analysis (based on three-fold cross-validation using the limma t-test)

Top 15 metabolite/lipid features
Lipids: –C**H**$_2$–CH=CH– in FAC and C**H**$_3$ in NAG
Lactate
Lipids: =CH–C**H**$_2$–CH= in FAC (a)
α-Ketoglutarate
Cysteine
Lipids:=CH–C**H**$_2$–CH= in FAC (b)
Acetoacetate
α-D-glucopyranose
Lysyl
Lipids: –CH=CH– in FAC
Proline
Alanine
Lipids: C**H**$_3$–(CH$_2$)$_n$– in FAC
Methionine
Asparagine

Abbreviations: FAC: fatty acid chain, NAG: N-acetylated glycoproteins

Table F Top integration regions for the PepsNMR pre-processed 900 MHZ spectral binning analysis (based on three-fold cross-validation using the limma t-test)

Top 15 integration regions	
Integration region	Signal found in region
[3.9680, 3.9600]	Creatine
[2.2680, 2.2563]	Acetone
[2.1930, 2.1700]	Glutamine
[3.8410, 3.8140]	Glucose
[2.6900, 2.6597]	Methionine
[3.9150, 3.8920]	Glucose
[3.9920, 3.9680]	Asparagine, Histidine, Serine, Tyrosine
[3.0940, 3.0785]	Tyrosine
[6.7600, 6.7004]	Not identified
[3.7240, 3.6500]	Glycerol
[0.9800, 0.9550]	Isoleucine
[1.3730, 1.3516]	Lactate
[3.6163, 3.5930]	Threonine
[7.3510, 7.3227]	Not identified
[2.1700, 2.1650]	Methionine

Table G Top metabolite/lipid features for the PepsNMR pre-processed 900 MHz BATMAN analysis (based on three-fold cross validation using the limma t-test)

Top 15 metabolite/lipid features
α-D-glucopyranose
α-Ketoglutarate
Serine
Histidine
Citrate
Glycine
Glutamate
Threonine
Tryptophan
Lipids: C**H**$_3$–(CH$_2$)$_n$– in FAC
Tyrosine
Myo-inositol
Lipids: –C**H**$_2$–C=O or –C**H**$_2$–CH=CH– in FAC
Lipids: =CH–C**H**$_2$–CH= in FAC (b)
Asparagine

Abbreviations: FAC: fatty acid chain

Table H Top integration regions for the manually pre-processed 900 MHz spectral binning analysis (based on three-fold cross-validation using the limma t-test)

Top 15 integration regions	
Integration region	Signal found in region
[3.7204, 3.6453]	Glycerol
[2.1289, 2.0993]	Lipids: : –C**H**$_2$–CH=CH– in FAC
[3.9120, 3.8957]	Glucose
[2.0993, 1.9889]	Lipids: : –C**H**$_2$–CH=CH– in FAC, C**H**$_3$ of NAG
[5.2186, 5.2038]	C$_2$H in glycerol backbone of PL and TG
[2.6768, 2.6597]	Methionine
[1.4587, 1.4201]	Lysine
[0.9766, 0.9663]	Isoleucine
[3.6163, 3.5861]	Threonine
[1.2240, 1.1766]	Not identified
[3.9903, 3.9644]	Asparagine, Histidine, Serine, Tyrosine
[7.0792, 7.0597]	Histidine
[6.7460, 6.7004]	Not identified
[1.0513, 1.0340]	Isoleucine
[3.6453, 3.6212]	Valine

Abbreviations: FAC: fatty acid chain, NAG: N-acetylated glycoproteins, PL: phospholipids, TG: triglycerides

Multivariate analysis

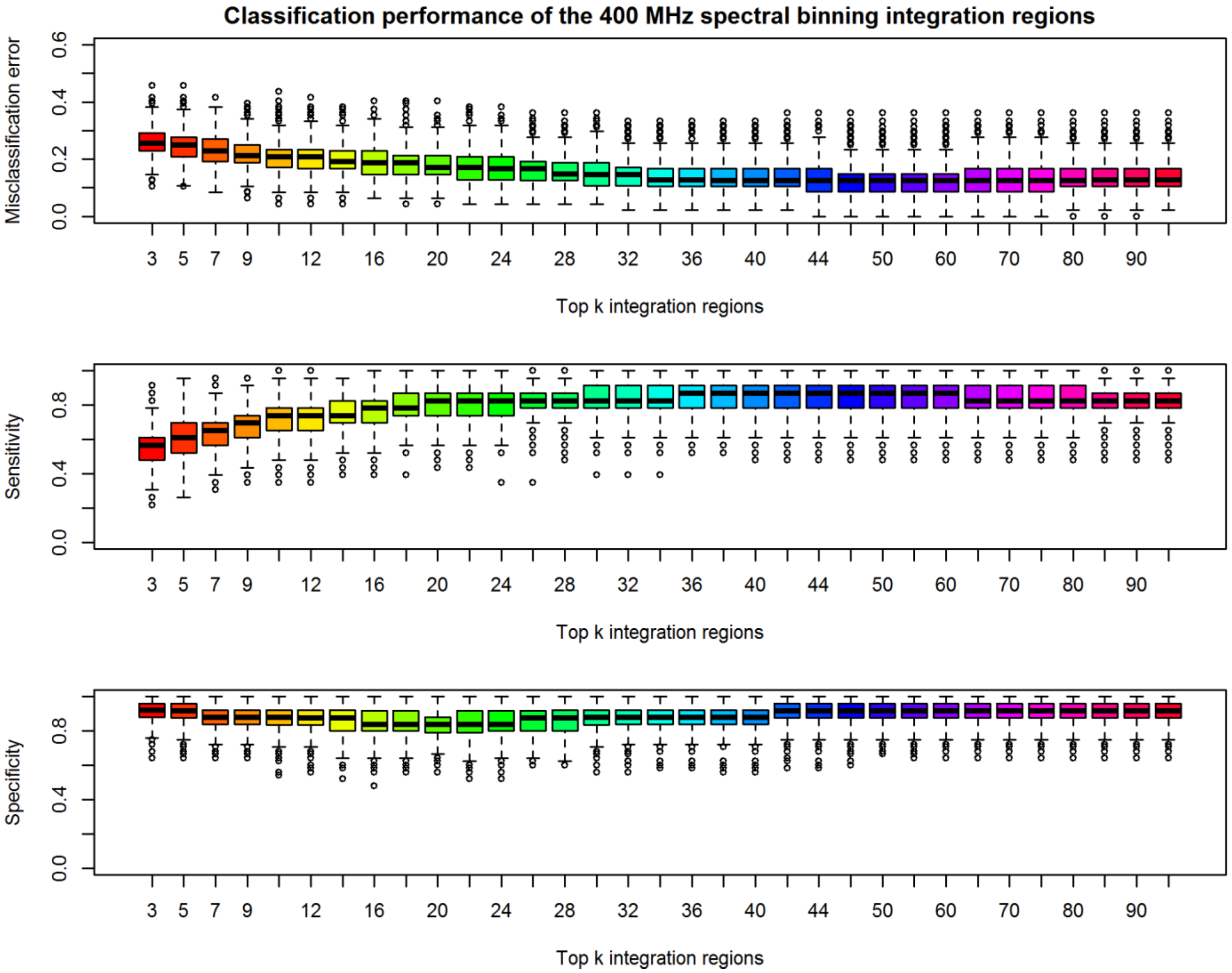

Figure G Classification performance of the elastic net models utilizing the top k 400 MHz spectral binning integration regions

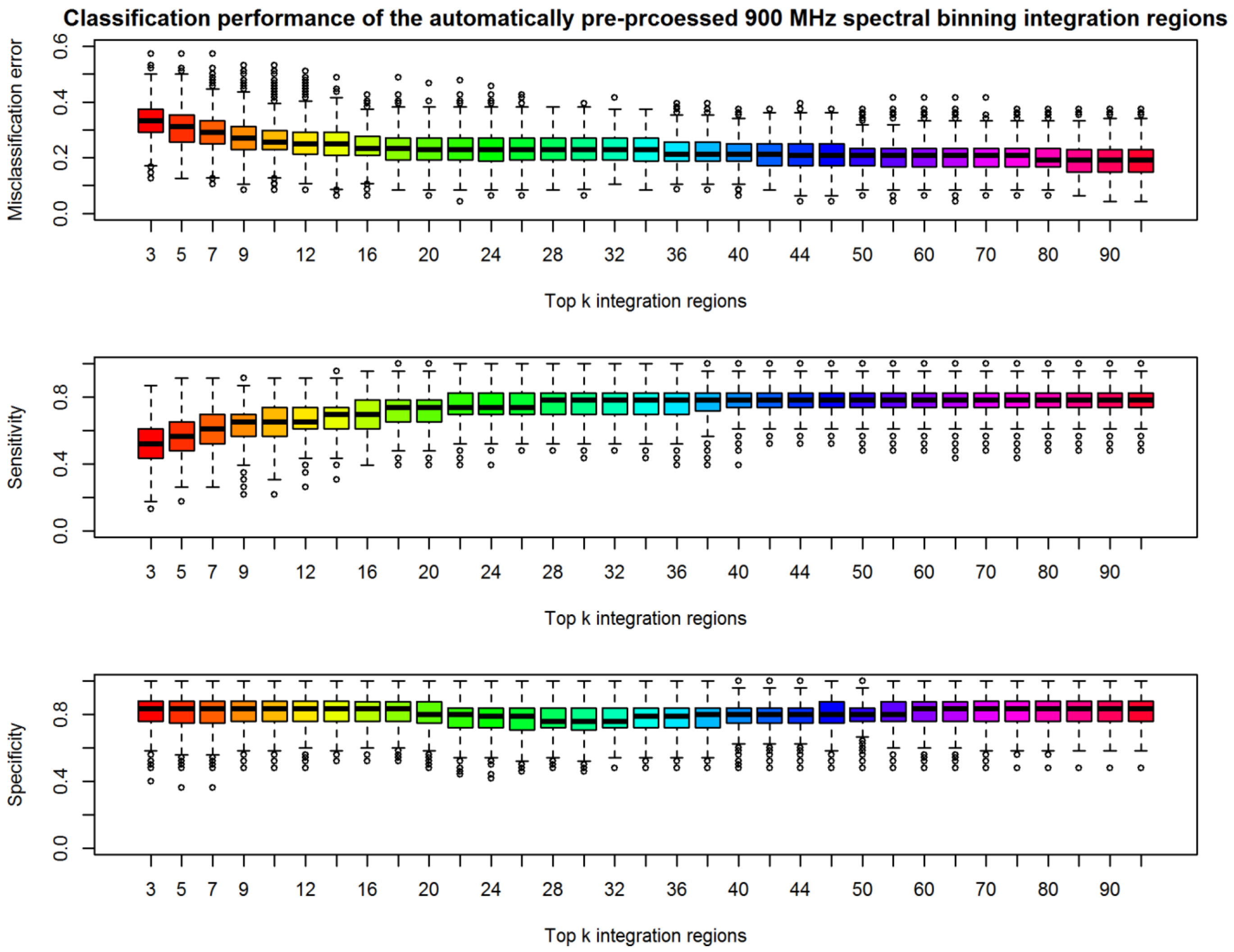

Figure H Classification performance of the elastic net models utilizing the top k PepsNMR pre-processed 900 MHz spectral binning integration regions

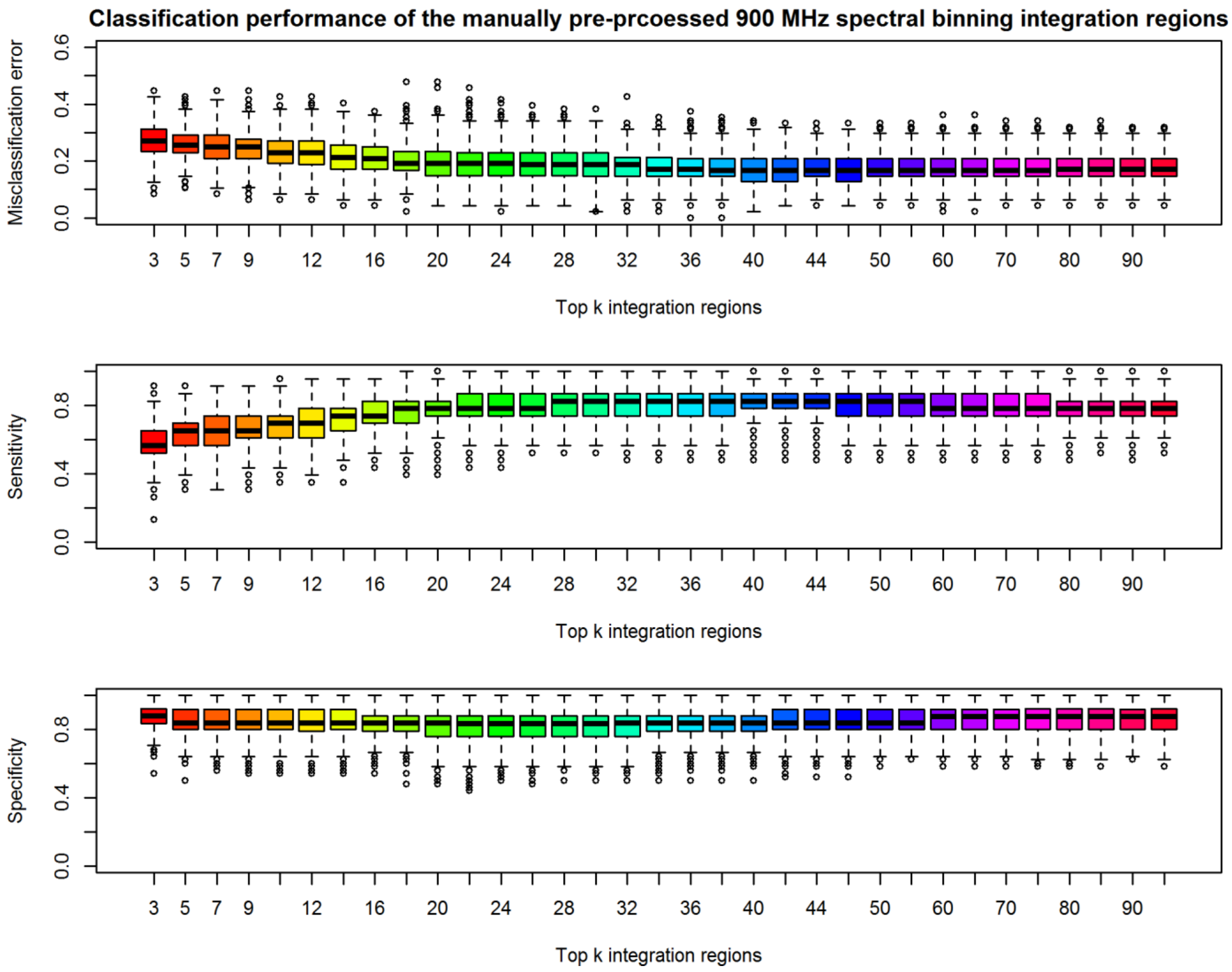

Figure I Classification performance of the elastic net models utilizing the top k manually pre-processed 900 MHz integration regions

Table I Classification results

Features	Classification error	Sensitivity	Specificity
Elastic net			
400 MHz (manually pre-processed data)			
Binning: top 50 integration regions	0.126 (0.002)	0.843 (0.003)	0.904 (0.002)
BATMAN: all metabolites	0.336 (0.002)	0.634 (0.003)	0.691 (0.003)
BATMAN: lipids	0.260 (0.002)	0.705 (0.003)	0.772 (0.003)
BATMAN: all metabolites and lipids	0.197 (0.002)	0.775 (0.003)	0.829 (0.002)
900 MHz (PepsNMR automatically pre-processed data)			
Binning: top 90 integration regions	0.197 (0.002)	0.779 (0.003)	0.826 (0.003)
BATMAN: all metabolites	0.105 (0.001)	0.884 (0.002)	0.906 (0.002)
BATMAN: lipids	0.323 (0.002)	0.611 (0.003)	0.738 (0.003)
BATMAN: all metabolites and lipids	0.111 (0.001)	0.874 (0.002)	0.902 (0.002)
900 MHz (manually pre-processed data)			
Binning: top 60 integration regions	0.169 (0.002)	0.804 (0.003)	0.857 (0.002)
Lasso			
400 MHz (manually pre-processed data)			
Binning: top 45 integration regions	0.136 (0.002)	0.825 (0.003)	0.901 (0.002)
BATMAN: all metabolites	0.333 (0.002)	0.632 (0.003)	0.700 (0.003)
BATMAN: lipids	0.261 (0.002)	0.704 (0.003)	0.771 (0.003)
BATMAN: all metabolites and lipids	0.197 (0.002)	0.768 (0.003)	0.834 (0.002)
900 MHz (PepsNMR automatically pre-processed data)			
Binning: top 90 integration regions	0.206 (0.002)	0.764 (0.003)	0.821 (0.003)
BATMAN: all metabolites	0.112 (0.001)	0.877 (0.002)	0.899 (0.002)
BATMAN: lipids	0.324 (0.002)	0.612 (0.003)	0.737 (0.003)
BATMAN: all metabolites and lipids	0.122 (0.001)	0.862 (0.002)	0.892 (0.002)
900 MHz (manually pre-processed data)			
Binning: top 45 integration regions	0.170 (0.002)	0.799 (0.003)	0.859 (0.002)
Orthogonal Partial Least Squares - Discriminant Analysis			
400 MHz (manually pre-processed data)			
Binning: top 60 integration regions	0.141 (0.002)	0.768 (0.003)	0.943 (0.002)
BATMAN: all metabolites	0.307 (0.002)	0.650 (0.003)	0.732 (0.003)
BATMAN: lipids	0.225 (0.002)	0.697 (0.003)	0.848 (0.002)
BATMAN: all metabolites and lipids	0.217 (0.002)	0.725 (0.003)	0.838 (0.003)
900 MHz (PepsNMR automatically pre-processed data)			
Binning: top 90 integration regions	0.193 (0.002)	0.746 (0.003)	0.863 (0.002)
BATMAN: all metabolites	0.109 (0.001)	0.883 (0.002)	0.898 (0.002)
BATMAN: lipids	0.253 (0.002)	0.714 (0.003)	0.778 (0.002)
BATMAN: all metabolites and lipids	0.103 (0.001)	0.891 (0.002)	0.903 (0.002)
900 MHz (manually pre-processed data)			
Binning: top 60 integration regions	0.199 (0.002)	0.697 (0.003)	0.897 (0.002)

Random forest			
400 MHz (manually pre-processed data)			
Binning: top 60 integration regions	0.160 (0.002)	0.805 (0.003)	0.872 (0.002)
BATMAN: all metabolites	0.362 (0.002)	0.584 (0.003)	0.689 (0.003)
BATMAN: lipids	0.279 (0.002)	0.689 (0.003)	0.750 (0.003)
BATMAN: all metabolites and lipids	0.265 (0.002)	0.703 (0.003)	0.765 (0.003)
900 MHz (PepsNMR automatically pre-processed data)			
Binning: top 80 integration regions	0.222 (0.002)	0.746 (0.003)	0.807 (0.003)
BATMAN: all metabolites	0.124 (0.001)	0.858 (0.002)	0.893 (0.002)
BATMAN: lipids	0.284 (0.002)	0.691 (0.003)	0.739 (0.003)
BATMAN: all metabolites and lipids	0.131 (0.002)	0.855 (0.002)	0.881 (0.002)
900 MHz (manually pre-processed data)			
Binning: top 45 integration regions	0.175 (0.002)	0.773 (0.003)	0.874 (0.002)
Support vector machines			
400 MHz (manually pre-processed data)			
Binning: top 90 integration regions	0.137 (0.001)	0.838 (0.003)	0.887 (0.002)
BATMAN: all metabolites	0.353 (0.002)	0.548 (0.005)	0.739 (0.004)
BATMAN: lipids	0.225 (0.002)	0.713 (0.003)	0.833 (0.002)
BATMAN: all metabolites and lipids	0.226 (0.002)	0.735 (0.003)	0.811 (0.003)
900 MHz (PepsNMR automatically pre-processed data)			
Binning: top 80 integration regions	0.213 (0.002)	0.759 (0.003)	0.813 (0.003)
BATMAN: all metabolites	0.142 (0.001)	0.850 (0.002)	0.866 (0.002)
BATMAN: lipids	0.246 (0.002)	0.699 (0.003)	0.806 (0.003)
BATMAN: all metabolites and lipids	0.117 (0.001)	0.879 (0.002)	0.887 (0.002)
900 MHz (manually pre-processed data)			
Binning: top 80 integration regions	0.177 (0.002)	0.785 (0.003)	0.858 (0.003)

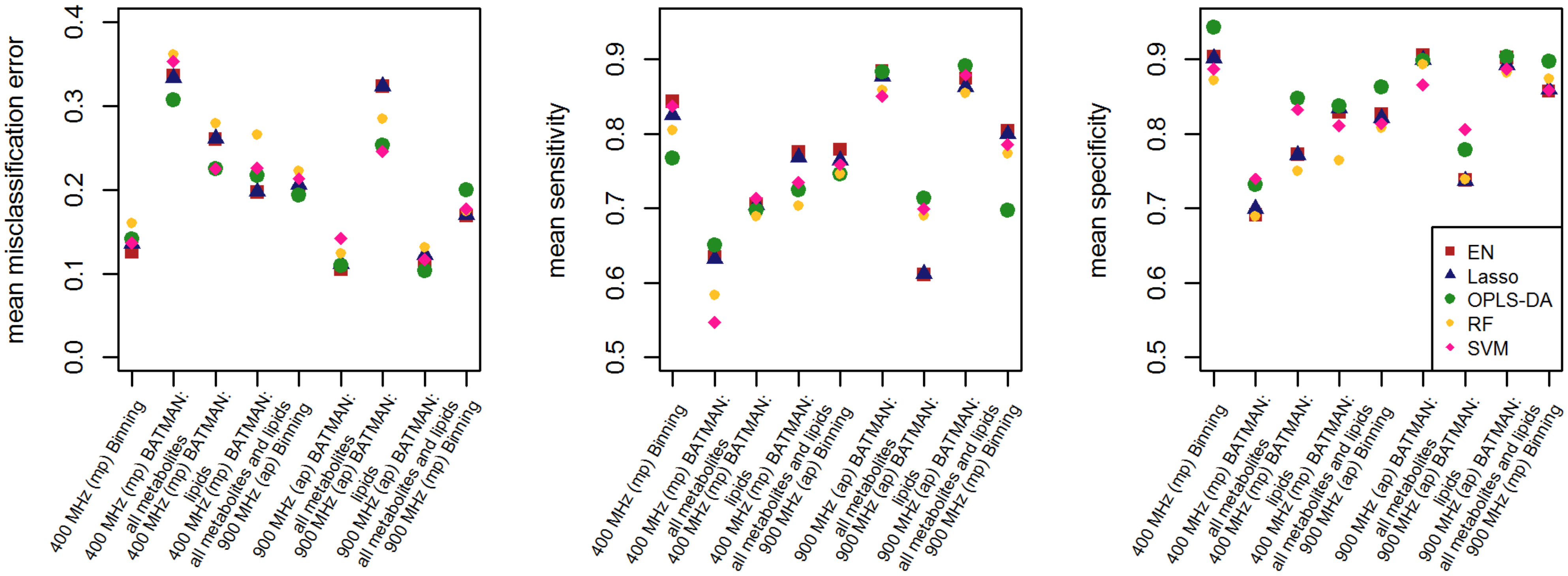

Figure J Classification performance in terms of mean misclassification error, mean sensitivity and mean specificity of the elastic net, lasso, orthogonal partial least squares-discriminant analysis (OPLS-DA), random forest (RF), and support vector machine (SVM)

References

[1] Hao Jie, Liebeke Manuel, Astle William, De Iorio Maria, Bundy Jacob G, Ebbels Timothy MD. Bayesian deconvolution and quantification of metabolites in complex 1D NMRspectra using BATMAN. Nature protocols. 2014;9(6):1416.

[2] Louis Evelyne, Bervoets Liene, Reekmans Gunter, et al. Phenotyping human blood plasma by 1H-NMR: a robust protocol based on metabolite spiking and its evaluation in breast cancer. Metabolomics. 2015;11(1):225–236.

[3] Hastie Trevor, Tibshirani Robert, Friedman Jerome. The elements of statistical learning. Springer; 2009.

[4] Bylesjö Max, Rantalainen Mattias, Cloarec Oliver, Nicholson Jeremy K, Holmes Elaine, Trygg Johan. OPLS discriminant analysis: combining the strengths of PLS-DA and SIMCA classification. Journal of Chemometrics. 2006;20(8-10):341-351.
